# Salvianolic acid B inhibits glycolysis in oral squamous cell carcinoma via targeting PI3K/AKT/HIF-1α signaling pathway

**DOI:** 10.1038/s41419-018-0623-9

**Published:** 2018-05-22

**Authors:** Jie Wei, Jun Wu, Wen Xu, Hong Nie, Ruiqing Zhou, Rui Wang, Yang Liu, Guoyao Tang, Jianyong Wu

**Affiliations:** 1 Department of Stomatology, Xin Hua Hospital, Shanghai Jiao Tong University School of Medicine, Shanghai, 200092 China; 2 Department of Laboratory Medicine, Shanghai General Hospital, Shanghai Jiao Tong University, Shanghai, 200080 China; 3 State Key Laboratory of Bioreactor Engineering and Shanghai Key Laboratory of New Drug Design, School of Pharmacy, East China University of Science and Technology, shanghai, 200237 China; 4 Shanghai Institute of Immunology, Department of Immunology and Microbology, Shanghai Jiao Tong University School of Medicine, Shanghai, 200025 China; 5 Shanghai Key Laboratory of New Drug Design, School of Pharmacy, East China University of Science and Technology, Shanghai, 200237 China; 6 Department of Somatology, Wuxi People’s Hospital, Nanjing Medical University, Wuxi, 214023 China

## Abstract

Our previous study demonstrated a progressive glycolytic perturbation during the course of DMBA-induced hamster oral carcinogenesis, which was attenuated by salvianolic acid B (Sal-B) treatment along with decreased incidences of oral squamous cell carcinoma (OSCC) formation. It was proposed that metabolic modulation should be an additional mode of action attributable to Sal-B’s anti-carcinogenic activity. However, the molecular mechanisms underlying Sal-B-induced metabolic modulation function remained elusive. In the present study, we performed next-generation sequencing (NGS) profiling in the same animal model and found Sal-B treatment evoked a general downregulation of the phosphatidylinositol-4,5-bisphosphate 3-kinase (PI3K) and hypoxia inducible factor 1α subunit (HIF-1α) signaling pathways, which might contribute to Sal-B’s metabolic modulation activity. The inhibitory effects of Sal-B on aerobic glycolysis, as well as PI3K/AKT and HIF-1α signaling pathways, were validated in two well-characterized OSCC cell lines (Cal27 and HN4), and premalignant oral Leuk1 cells and Sal-B treatment led to elevation of the loss of mitochondrial membrane potential (MMP), increased cell apoptosis, and reduced abilities of colony formation. Rescue assays suggested that compared with Sal-B treatment group, Akt or hif-1a overexpression attenuated the inhibitory effect of Sal-B on glucose uptake and intracellular lactate level. Taken together, our results suggested that Sal-B modulated aberrant glucose metabolism via the PI3K/AKT/HIF-1α signaling pathways, which might contribute to the anti-carcinogenic activity of Sal-B.

## Introduction

Oral squamous cell carcinoma (OSCC) is currently the sixth most common malignancy worldwide and ranks eighth in cancer-related mortalities^1^. Despite some progress achieved during the last decades in its diagnostics and therapeutic options, the 5-year survival of OSCC has remained at 50–60%, largely unchanged for 40 years^2,3^. OSCC has a well-characterized progression from hyperplasia through dysplasia to carcinoma with a multistep process involving the accumulation of numerous genetic and epigenetic in oncogenes and suppressor genes, leading to dysregulation of multiple signaling pathways, which disrupt the cell cycle and the balance between cell proliferation and cell death^4^. It usually takes many years for normal epithelial cells to undergo the multiple cellular and genetic alterations that lead to malignant changes, making OSCC an optimal disease for pharmacological interventions before cancer transformation^4^.

Chemoprevention has been considered a rational and appealing strategy to prevent or delay the development of OSCC. Previously, the most extensive clinically studied trials have utilized local deliveries with several classes of compounds such as vitamin A (or retinlo), 13-*cis* retinoic acid, cyclooxygenase-2 inhibitors, and chemotherapy agents^5–8^. However, these supplements may actually lead to unexpected detrimental effects and the beneficial effects have been infrequent or transient, limiting the extensive and chronic use of these drugs. This challenging clinical scenario indicates the need for better effective, nontoxic, and affordable novel chemopreventive agents in the management oral carcinogenesis. Salviae miltiorrhizae (Danshen or Tanshen) has been widely used in traditional Chinese medicine practice for the treatment of cardiovascular and cerebrovascular diseases with minimal side effects^9^. Salvianolic acid B (Sal-B), the most abundant and bioactive water-soluble compound of *Salviae miltiorrhizae*, has been reported to inhibit chemically induced oral carcinogenesis in multiple studies^10^. Angiogenesis may be one of the possible mechanisms behind the preventive effects. Besides, Sal-B might intervene the malignant conversion via its anti-cancer properties including cell cycle arrest, induction of apoptosis, inhibition of oxidation, and inflammation, etc.^11–13^.

Current insights into tumor biology promoted that metabolic reprogramming is a hallmark of cancer^14^. We previously performed a metabonomic study on the classical model of 7,12-dimethylbenz(a)anthracene (DMBA)-induced oral carcinogenesis and revealed significant alterations of key metabolic pathways correlated with disease progression, indicating a potential role of atypical metabolism in oral carcinogenesis^15^. Sal-B significantly attenuated the metabolic alterations, which was consistent with its beneficial effects that markedly inhibited incidences of OSCC formation. It was proposed that metabolic modulation should be an additional mode of action attributable to Sal-B’s anti-carcinogenic activity. However, the molecular mechanisms underlying Sal-B-induced metabolic modulation function remained elusive. In the present study, we performed next-generation sequencing profiling in the same animal model with the aim of specifically filling the knowledge gaps, followed by functional verification of the results. We believe this study would enhance our knowledge of the pathogenesis of this malignancy and potentially aid in elucidating the mechanisms of action of Sal-B in OSCC.

## Results

### Analysis based on the sequencing data

**Fig. 1:**
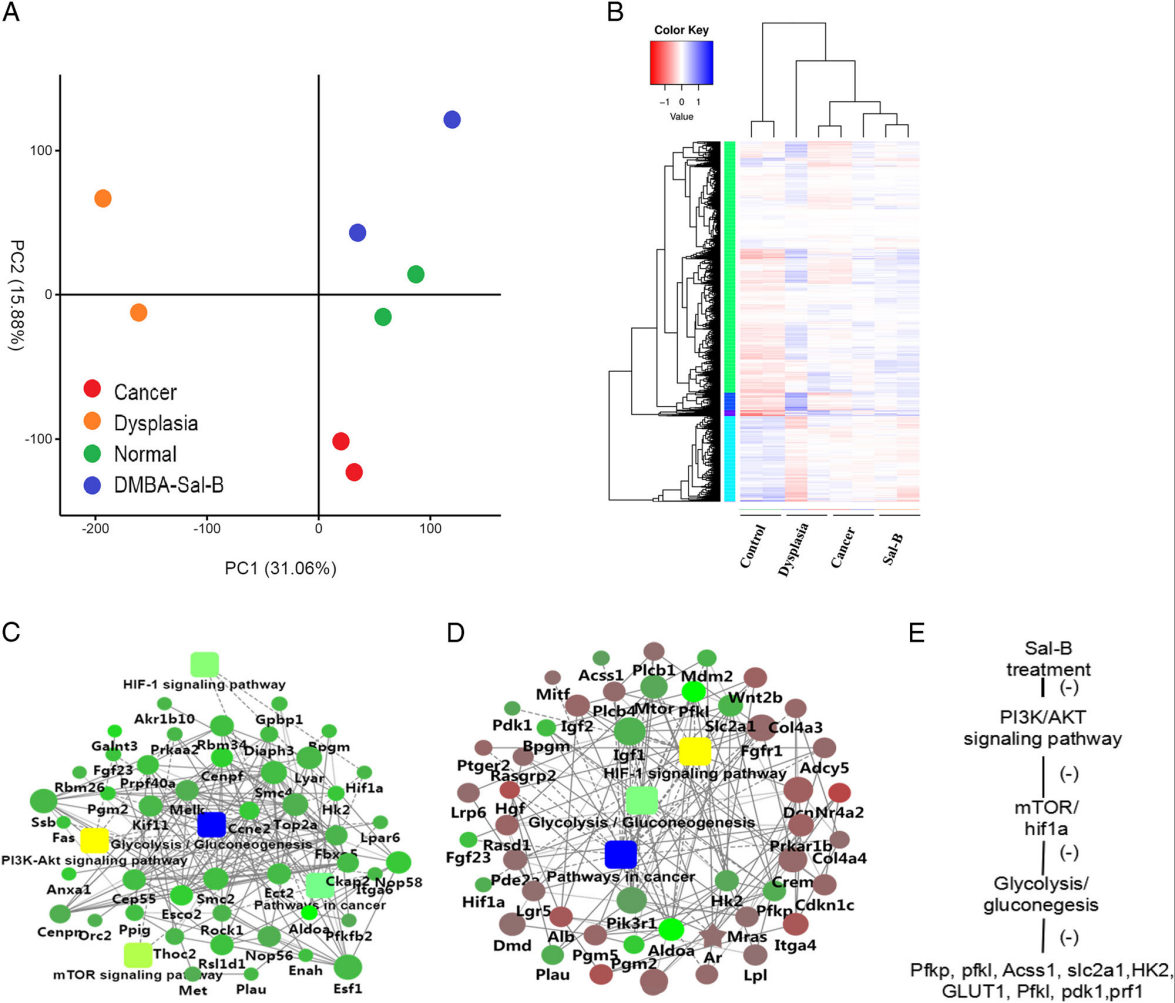
Transcriptional landscape for DMBA-induced model and Sal-B-DMBA-treated animal model. **a** PCA of the entire set of expressed genes. **b** Heatmap of gene expression data are shown for all samples (columns) color-coded (bottom of heatmap) the same as the PCA plot. Data were log2 transformed and then normalized before generating heatmap for direct comparison of data. DEGs (red or blue are downregulated or upregulated, respectively) for each animal were mapped by lane for each of two samples from the normal control group, DMBA-induced model group and Sal-B–DMBA group. **c**, **d** Gene interaction network of downregulated genes in Sal-B–DMBA-treated group versus DMBA-induced dysplasia (**c**) or cancer group (**d**). The networks were clustered into modules and pathways enriched in the modules are indicated. Circle nodes for genes/proteins; rectangle for KEGG pathway or biological process. Pathway were colored with gradient color from yellow to blue, yellow for smaller *P*-value, blue for bigger *P*-value. Biological processes were colored with red. In case of fold-change analysis, genes/proteins were colored in red (upregulation) and green (downregulation). **e** The predicted molecular action of Sal-B. DEGs, differentially expressed genes; PCA, principal component analysis

Previously, we have successfully established the DMBA-induced oral carcinogenesis model and Sal-B treatment dramatically decreased the oral cancer formation, whereas it did not prevent dysplasia^15^. Such observation inspired us to understand the protective role of Sal-B in the OSCC. Representative cheek pouches from the normal control group, DMBA-induced model group and Sal-B–DMBA-treated group were selected and sequenced. The principal component analysis (PCA) showed that the normal samples were differentially located at the PC2 axis, whereas the other samples did not form very clearly separate clusteres despite one sample in the Sal-B–DMBA-treated group located far away (Fig. 1a). We performed a hierarchical clustering analysis to determine whether these groups were molecularly distinct. As displayed on the heatmap, the normal control group showed distinguishable gene patterns, whereas the other three groups had relatively close relationships and were not distinctly distinguishable from one another with partial overlap (Fig. 1b). In spite of that, the patterns of Sal-B–DMBA-treated group were more similar to that of dysplasia rather than the cancer group, which paralleled with our early finding that Sal-B treatment inhibited tumor development of OSCC but did not prevent dysplasia.

**Fig. 2:**
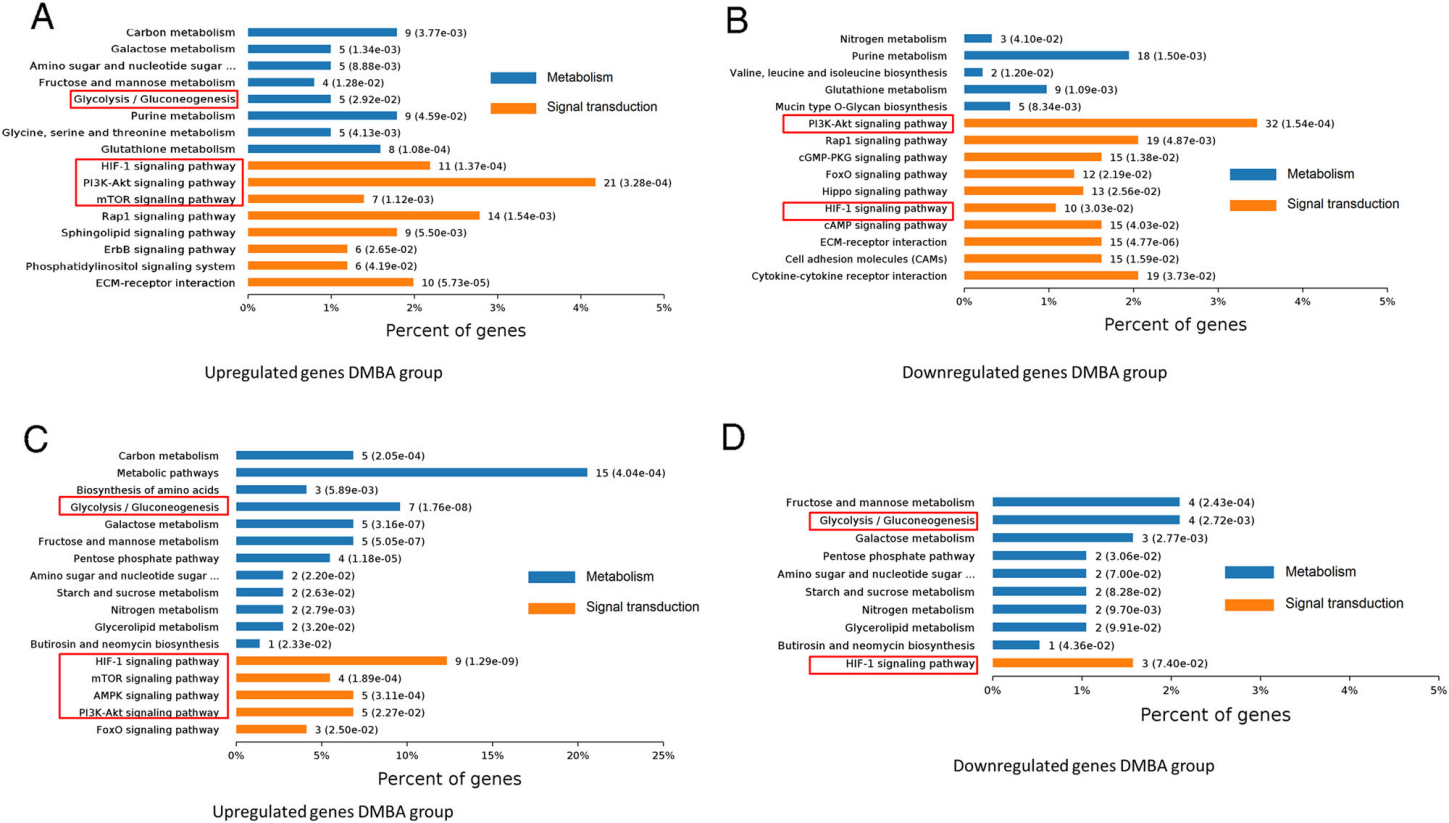
Pathway analyses of DEGs in specified comparisons. **a** Pathways significantly enriched in upregulated genes in DMBA-induced cancer vs. normal control. **b** Pathways significantly enriched in upregulated genes in DMBA-induced dysplasia versus normal control. **c** Pathways significantly enriched in downregulated genes in Sal-B-DMBA-treated group vs. DMBA-induced cancer. **d** Pathways significantly enriched in downregulated genes in Sal-B-DMBA-treated group vs. DMBA-induced dysplasia. The red frame indicated the significantly related pathways in this study

We performed six comparisons for a total of four groups and six volcano figures were shown in Supplementary Figure 1A. The overlaps in differentially expressed genes (DEGs) for each comparison were shown in Supplementary Figure 1B. The identified DEGs in the six comparisons were enriched by Gene Ontology (GO) and Kyoto Encyclopedia of Genes and Genomes (KEGG) pathway analyses (top ten GO and pathway terms in each comparison were listed in Supplementary Table 1 and Table 2). To overview the disease-related processes, we compared the expression profile data for comparisons between DMBA-induced model and the normal control group. Enrichment analysis of signaling pathways mediated by the DEGs upregulated in DMBA-induced dysplasia or cancer group showed that numerous pathways were activated in the initial and late phases of carcinogenesis, including the potentially oncogenic hypoxia inducible factor 1α subunit (HIF-1α), mammalian target of rapamycin (mTOR), and phosphatidylinositol-4,5-bisphosphate 3-kinase (PI3K)/Akt signaling pathways, which have been reported to have pivotal roles in cancer initiation and progression (Fig. 2a, b). Galactose metabolism and glycolysis/gluconeogenesis were enriched and the latter ranked highest significance in dysplasia group and did not deteriorate as disease progression, implicating that glucose metabolism disordered in early pathogenesis of OSCC. We next focused on pathways changed by Sal-B treatment. Network and pathway analyses revealed that Sal-B treatment evoked a general downregulation of glycolysis/gluconeogenesis, metabolic pathways, and HIF-1α signaling pathways in DMBA-induced model group (Fig. 1c, d and Fig. 2c, d). HIF-1α is a transcriptional factor elevated under aerobic conditions and the levels of its activity have already been reported to implicate in tumorigenicity, angiogenesis, and also glycolysis. It was downstream from activated PI3K and the latter closely linked to the pathogenesis of OSCC. Above all, elevated glucose metabolism occurred even before malignant transformation of OSCC and Sal-B modulated aberrant glucose metabolism via the PI3K/AKT/HIF-1α signaling pathways, which might contribute to the anti-carcinogenic activity of Sal-B (Fig. 1e).

### Sal-B inhibited PI3K/Akt/HIF-1α pathway and glycolysis in the animal model

**Fig. 3:**
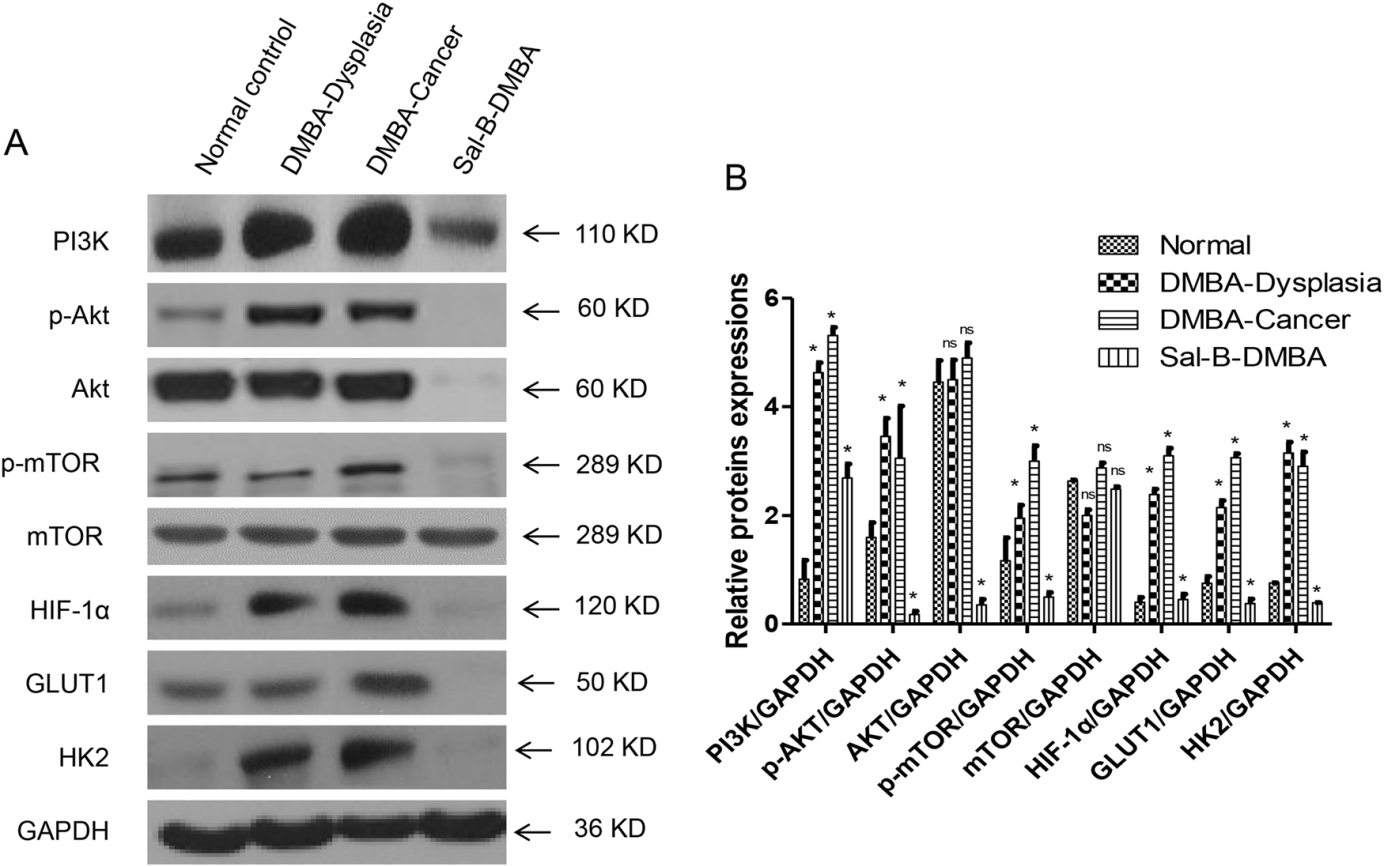
Sal-B inhibits PI3K/Akt and HIF-1α signaling and glycolysis in the animal model. **a** Western blot analysis of cheek pouches from DMBA-induced animal model. PI3K and its key downstream proteins (Akt, p-Akt, mTOR, and p-mTOR), HIF-1α, and glycolytic-related proteins (GLUT1 and HK2) were detected. **b** Quantitive analysis from (**a**). *P* < 0.05 was considered statistically significant. *p < 0.05 vs. control group, ns, *P* > 0.05

As it has been postulated that Sal-B modulated glucose metabolism via PI3K/AKT/HIF-1α signaling pathways, we further validated the results with western blotting in the animal model. PI3K and its downstream AKT, p-AKT, and p-mTOR expression levels were significantly elevated in DMBA-induced model group compared with normal mucosal group, which were dramatically reduced by Sal-B treatment (Fig. 3). In addition, there was no significant difference in the expression levels of these proteins between the tissue specimens of DMBA-induced dysplasia and cancer (Fig. 3). Similarly, upregulated HIF-1α was observed in DMBA-induced model group and was significantly suppressed by Sal-B treatment (Fig. 3).

Glucose transporter 1 (GLUT1) is a transmembrane glucose transport protein that allows the facilitated transport of glucose into cells^16^. Hexokinase 2 (HK2) is the enzyme controlling the first step of glycolysis and is overexpressed in a variety of malignant tumors^17^. Western blotting was employed to detect the expression level of GLUT1 and HK2 as indicators of glycolysis. Compared with normal mucosa, a significantly increased expression of GLUT1 and HK2 was observed in DMBA-induced model group and was remarkably attenuated by Sal-B treatment (Fig. 3). These results validated that Sal-B inhibited PI3K/AKT/HIF-1α signaling and glycolysis in the animal model.

### Sal-B inhibited PI3K/AKT/HIF-1α pathway and glycolysis in vitro

**Fig. 4:**
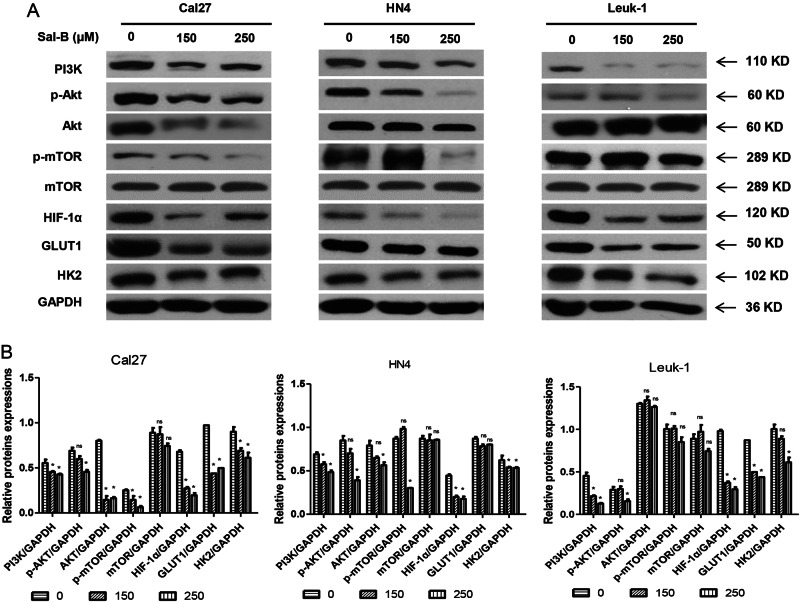
Sal-B inhibits PI3K/Akt/HIF-1α signaling and glycolysis in vitro. Cal27, HN4, and Leuk1 cells were treated with different concentrations of Sal-B for 48 h and detected by western blotting. **a** PI3K and its key downstream proteins (Akt, p-Akt, mTOR, and p-mTOR), HIF-1α, and glycolytic-related proteins (GLUT1 and HK2) were detected by western blotting. **b** Quantitive analysis from (**a**). *P* < 0.05 was considered statistically significant. *p < 0.05 vs. control group, ns, *P* > 0.05

We examined whether Sal-B treatment inhibited PI3K and its key downstream proteins, HIF-1α, and glycolysis in vitro. Two well-characterized OSCC cell lines Cal27 and HN4, and immortalized oral leukoplakia Leuk1 cells were treated with Sal-B at different concentrations for 48 h and subsequently evaluated by western blot assays. As shown in Fig. 4, we found Sal-B treatment significantly inhibited PI3K, Akt, phospho-Akt (p-Akt), and p-mTOR in Cal27 in a dose-dependent manner. Cells treated with Sal-B also showed a tendency of downregulation of HIF-1α compared with the vehicle group. Similarly, Sal-B treatment inhibited GLUT1 and HK2 expression in OSCC cells and premalignant leukoplakia cells. Similar results were obtained in HN4 and Leuk1. These results suggested that Sal-B inhibited PI3K and its key downstream proteins, HIF-1α, and glycolysis in vitro.

### Sal-B regulated glucose metabolism and lactate secret

**Fig. 5:**
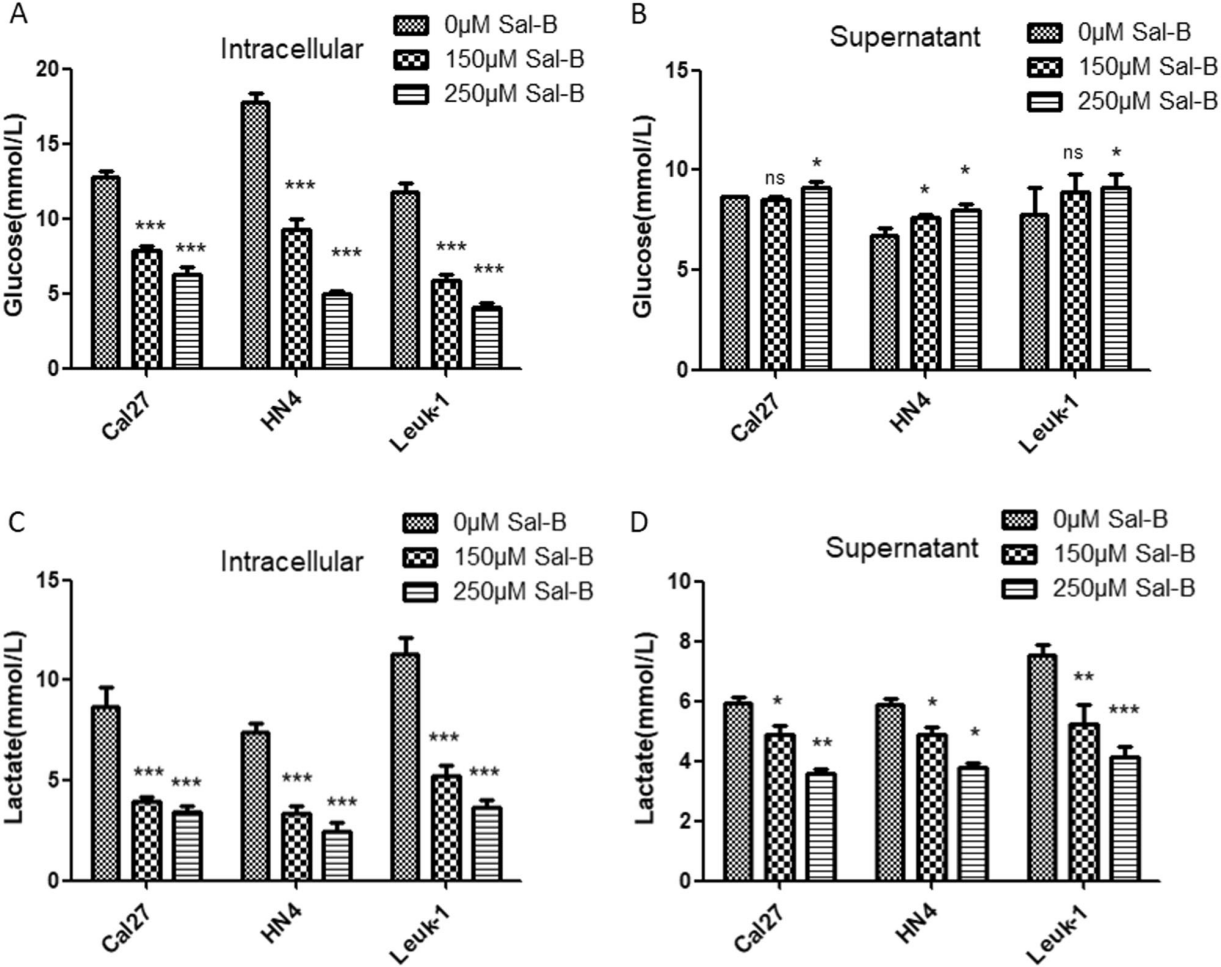
Sal-B inhibited the aberrant glucose metabolism. (**A,B**) Measurement of glucose intracellular and in supernatants treated with 0, 150, and 250 uM of Sal-B. (**C,D**) Measurement of lactate intracellular and in supernatants treated with 0, 150, and 250 uM of Sal-B. *P*<0.05 was considered statistically significant, ns *P*>0.05, *P<0.05, **P<0.01, ***P<0.001 vs control group.

We measured the glucose uptake and the lactate production in culture media of Sal-B-treated OSCC cells. As shown in Fig. 5a, b, the intracellular glucose in HN4, Cal27, and Leuk1 cells were gradually decreased after Sal-B treatment, and the supernatant glucose increased somewhat, which indicated that Sal-B reduce the glucose uptake in three cell lines. Consistently, lactate production significantly reduced from the supernatant and the intracellular cells after Sal-B treatment (Fig. 5).

### Sal-B inhibited cell proliferation and colonigenic ability

**Fig. 6:**
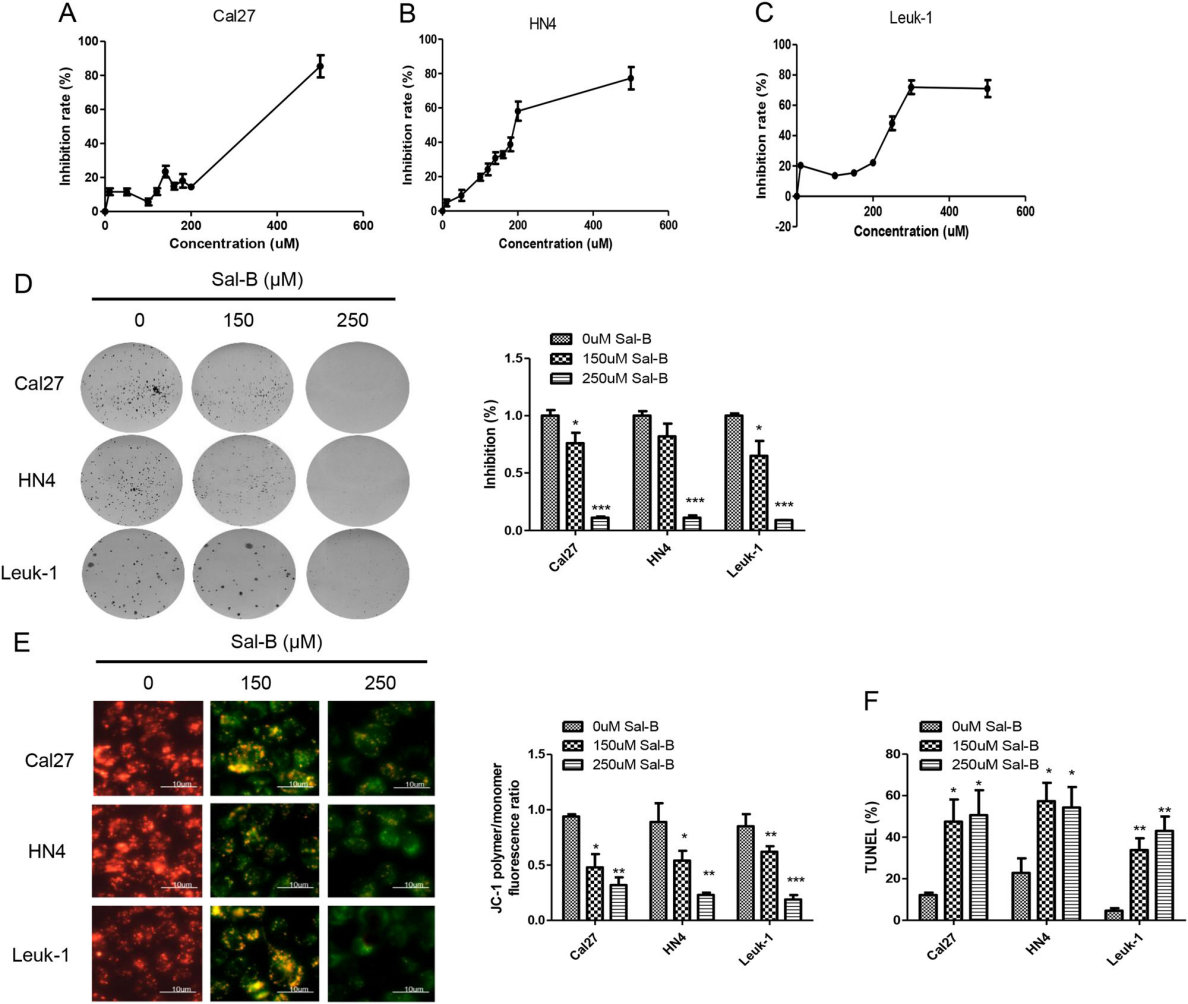
Sal-B inhibited proliferation and colonigenic ability and induced cell apoptosis. **a** Cal27, **b** HN4, and **c** Leuk1 cells were treated with Sal-B at different concentrations for 48 h and subsequently evaluated by the MTT assays. IC 50 values were 297 μM in Cal27, 201 μM in HN4, and 294 μM Leuk1, respectively. **d** Anchorage-independent growth via colony formation assay. Cal27, HN4, and Leuk1 cells were treated with 150 μm and 250 μM for 24 h, and subsequently evaluated by clony formation assay. **e** Quantitive analysis from (**d**). **e** MMP was measured by JC-1 staining in Cal27, HN4, and Leuk1 cells treated with 0, 150, and 250 μM of Sal-B. The fluorescence ratios of JC-1 ploymer to monomer were further quantified. Bar stands for 10 μm. **f** Apoptotic cell death of cells with treatment of 0, 150, and 250 μM of Sal-B measured by TUNEL assay. *P* < 0.05 was considered statistically significant, ns, *P* > 0.05, *P < 0.05, ** *P* < 0.01, *** *P* < 0.001 vs. control group

To validate the anti-proliferative effects of different doses of Sal-B, these cell lines were treated with Sal-B at different concentrations for 48 h and subsequently evaluated by the MTT (3-(4,5-dimethylthiazol-2-yl)-2,5-diphenyltetrazolium bromide) assays. Exposure to Sal-B resulted in a dose-dependent inhibition of cell viability with IC 50 values of 297 μM in Cal27, 201 μM in HN4, and 294 μM Leuk1 (Fig. 6a–c). We further examined the effect of Sal-B on colony formation. As shown in Fig. 5d, all these cell lines formed clonies on with saline treatment, whereas over 70% inhibition occurred with a dose of 150 μM Sal-B treatment in Cal27 cells and ~ 80% inhibition in HN4 cells. Similar results were obtained from the assays on Leuk1 cells. Sal-B (250 μM) treatment almost completely blocked the colony formation of these cell lines (Fig. 6d). These results revealed that Sal-B could induce growth inhibition both in OSCC cell lines and premalignant cells, suggesting that Sal-B is a potential modality for preventing and treating OSCC.

### Sal-B increased the loss of MMP and induced apoptosis in OSCC cells

Tumor cells exhibit high levels of glycolysis; however, mitochondria remain the major source of cellular energy in most cancer cell lines and tissues, which is also vital for tumor growth^14^. To investigate whether Sal-B affected mitochondria function, changes in mitochondrial membrane potential (MMP) were monitored by JC-1 staining. As shown in Fig. 6e, in OSCC cells, Sal-B treatment was able to increase the loss of MMP, demonstrated by remarkable reduced ratio of JC-1 polymer to monomer fluorescence, in a dose-dependent manner. To investigate the potential mechanism of cell death induced by Sal-B in this study, apoptosis was measured by the terminal deoxynucleotidyltransferase-mediated dUTP-biotin nick end labeling (TUNEL) assays. Consistently, cells also displayed increased apoptosis in response to Sal-B treatment in a dose-dependent manner compared with untreated cells (Fig. 6e, f and Figure S2). Our data indicated that Sal-B increased the collapse of the inner mitochondrial membrane and led to mitochondrial dysfunction, which lead to cause apoptosis.

### Sal-B regulated glycolysis via the activation of PI3K/Akt signaling pathway

**Fig. 7:**
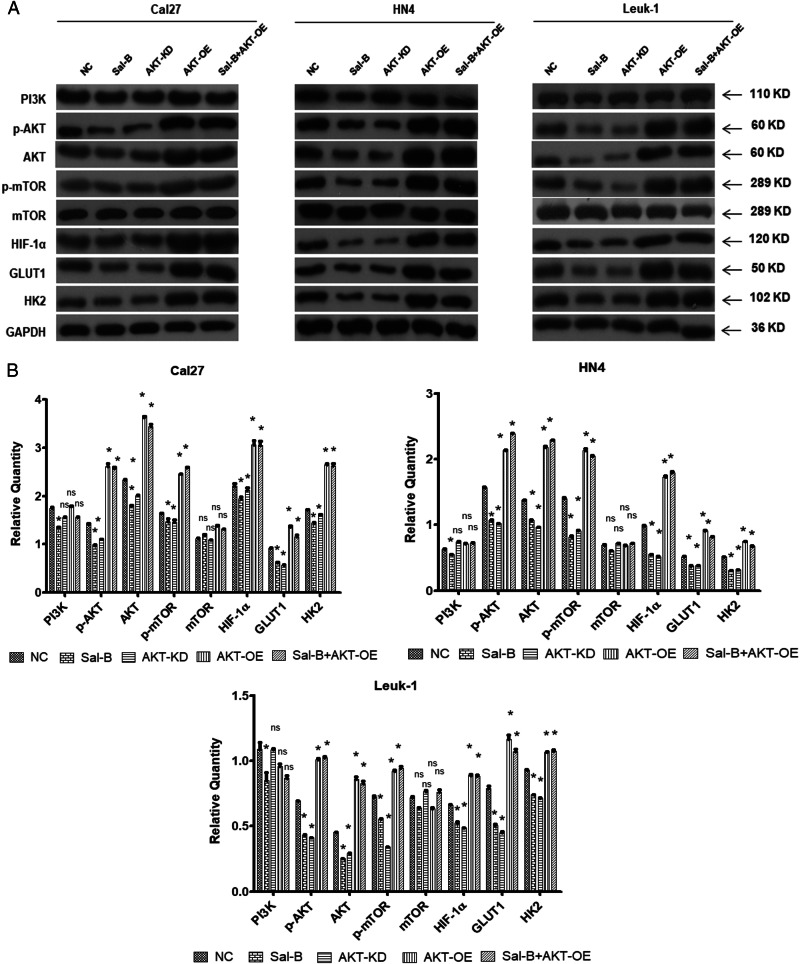
Sal-B inhibited PI3K/Akt/HIF-1α signaling and glycolysis in vitro. Cal27, HN4, and Leuk1 cells were treated with varying conditions, including (1) without treatment (NC), (2) 250 μM of Sal-B, (3) knockdown of AKT expression using AKT shRNA lentivirus (AKT-KD), (4) overexpression of AKT using transfection of AKT lentivirus (AKT-OE), and (5) the combined treatment of Sal-B and AKT-OE. **a** The quantitative analysis of the protein level of PI3K, p-AKT, AKT, p-mTOR, mTOR, HIF-1α, GLUT1, and HK2 in three types of cells (Cal27, HN4, and Leuk1) collected at 48 h post treatments were detected by western blotting. **b** Quantitive analysis from (**a**). *P* < 0.05 was considered statistically significant, ns, *P* > 0.05, *P < 0.05, **P < 0.01, ***P < 0.001 vs. control group

We identified that Sal-B-treated cells showed a reduced expression of PI3K and its downstream Akt, p-Akt, and p-mTOR. Further, decreased expressions of HIF-1α, GLUT1, and HK2 were revealed in Sal-B-treated cells. To further define the signal pathway of the anti-tumor role of Sal-B in OSCC cells, rescue assay was performed. When overexpressing AKT, the inhibitory activity of Sal-B on p-AKT, hif1-α, GLUT1, and HK2 were attenuated in three cell lines, which indicated that Sal-B might modulate the cell growth via PI3K/AKT pathway (Fig. 7).

**Fig. 8:**
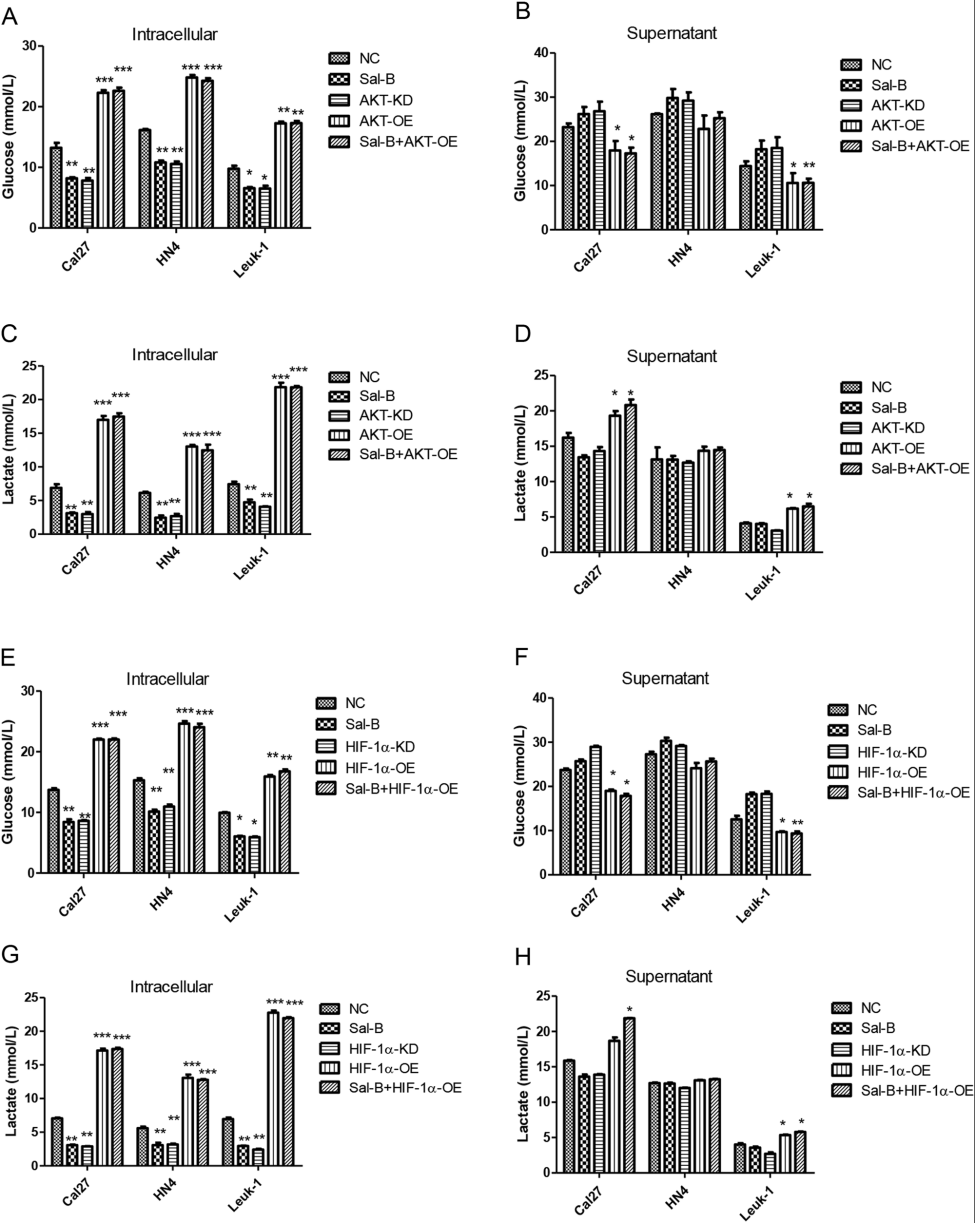
Sal-B modulates glucose metabolism via the activation of PI3K/Akt signaling pathway. Cal27, HN4, and Leuk1 cells were treated with varying conditions, including (1) without treatment (NC), (2) 250 μM of Sal-B, (3) knockdown of AKT expression using AKT shRNA lentivirus (AKT-KD), (4) overexpression of AKT using transfection of AKT lentivirus (AKT-OE), and (5) the combined treatment of Sal-B and AKT-OE. **a** Measurement of intracellular glucose in cells treated in varying conditions described above. **b** Measurement of glucose uptake in supernatants in cells treated in varying conditions described above. **c** Measurement of intracellular lactate production in cells treated in varying conditions described above. **d** Measurement of lactate production in supernatants from cells treated in varying conditions described above. Sal-B inhibit activation of HIF-1α signaling pathway to further modulate the glucose metabolism, elevated the loss of MMP and induces apoptosis. Cal27, HN4 and Leuk1 cells were treated with varying conditions, respectively, including (1) without treatment (NC), (2) 250 μM of Sal-B, (3) knockdown of HIF-1α expression using Hif-1α shRNA lentivirus (HIF-1α-KD), (4) overexpression of HIF-1α using transfection of Hif-1α lentivirus (HIF-1α-OE), and (5) the combined treatment of Sal-B and HIF-1α. **e** Measurement of intracellular glucose in cells treated in varying conditions described above. **f** Measurement of glucose uptake in supernatants in cells treated in varying conditions described above. **g** Measurement of intracellular lactate production in cells treated in varying conditions described above. **h** Measurement of lactate production in supernatants from cells treated in varying conditions described above. *P* < 0.05 was considered statistically significant, ns, *P* > 0.05, *P < 0.05, **P < 0.01, ***P < 0.001 vs. control group

Furthermore, Akt overexpression in Cal27, HN4, and Leuk1 cells induced remarkable high level of glucose uptake within the cells (Fig. 8a) and significantly suppressed level of glucose secretion to culture supernatant (Fig. 8b). Similarly, we also found that cells with Akt overexpression induced an increment of intracellular lactate from all cells we tested (Fig. 8c), whereas the lactate from the supernatant in both of Cal27 and Leuk1 cells treated with overexpression of Akt was also significantly elevated (Fig. 8d). In addition, when Hif-1α was overexpressed in Cal27, HN4, and Leuk1 cells, the similar trend as Akt overexpression was found (Fig. 8e–h and Figure S6). Taken together, compared with Sal-B treatment group, Akt or Hif-1α overexpression reduced the inhibitory effect of Sal-B on glucose uptake and intracellular lactate level, which indicated that Sal-B inhibited glucose metabolism via Akt/Hif-1α pathway.

### Sal-B enhanced cellular apoptosis by suppression of MMP via inhibiting the activation of PI3K/Akt/Hif-1α signaling pathway

**Fig. 9:**
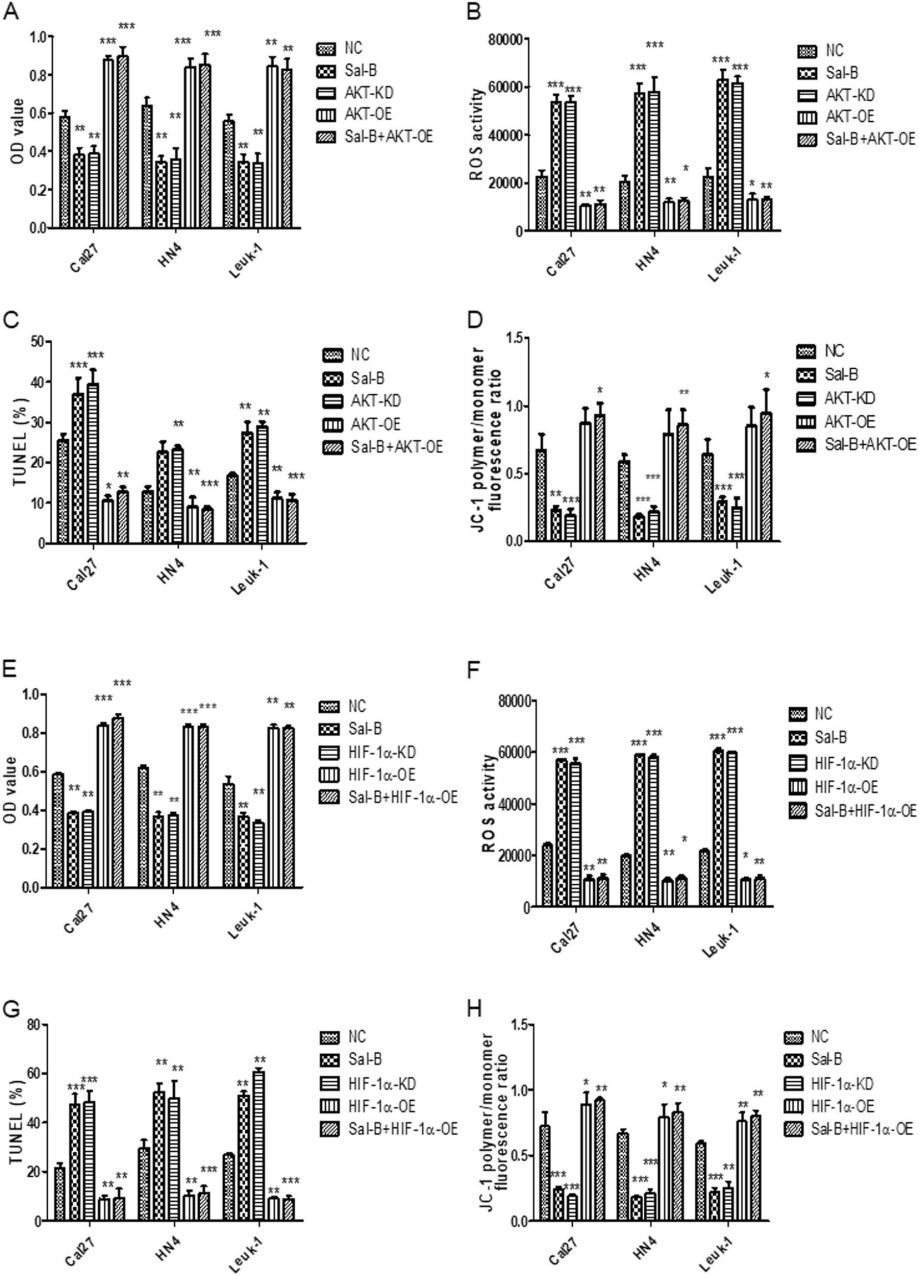
Sal-B suppresses the cellular proliferation, enhances the ROS activity and cellular apoptosis via inhibition of PI3K/AKT/HIF-1α signling pathway. **a** Cal27, HN4, and Leuk1 cells were treated with varying conditions, respectively, including (1) without treatment (NC), (2) 250 μM of Sal-B, (3) knockdown of AKT expression using AKT lentivirus (AKT-KD), (4) overexpression of AKT using transfection of plasmid AKT shRNA lentivirus (AKT-OE), and (5) the combined treatment of Sal-B and AKT-OE. The cellular proliferation of cells under varying conditions described above, revealed by MTT assay. **b** ROS activities of cells under varying conditions described in (**a**). **c** The cellular apoptosis of cells under varying conditions described in (**a**), demonstrated by TUNEL assay. **d** MMP was measured by JC-1 staining in Cal27, HN4, and Leuk1 cells under varying conditions described in (**a**). The fluorescence ratios of JC-1 ploymer to monomer were further quantified. Cal27, HN4, and Leuk1 cells were treated with varying conditions, respectively, including (1) without treatment (NC), (2) 250 μM of Sal-B, (3) knockdown of HIF-1α expression using Hif-1α shRNA lentivirus (HIF-1α-KD), (4) overexpression of HIF-1α using transfection of Hif-1α lentivirus (HIF-1α-OE), and (5) the combined treatment of Sal-B and HIF-1α. **e** The cellular proliferation of cells under varying conditions described above, revealed by MTT assay. **f** ROS activities of cells under varying conditions described in (**e**). **g** The cellular apoptosis of cells under varying conditions described in (**e**), demonstrated by TUNEL assay. **h** MMP was measured by JC-1 staining in Cal27, HN4, and Leuk1 cells under varying conditions described in **e**. The fluorescence ratios of JC-1 ploymer to monomer were further quantified. *P* < 0.05 was considered statistically significant, ns, *P* > 0.05, *P < 0.05, **P < 0.01, ***P < 0.001 vs. control group

As Sal-B was able to inhibit glucose uptake and lactate production through PI3K/Akt signaling pathway, we further demonstrated whether Sal-B-induced apoptosis also is partially attributed by activation of PI3K/Akt/Hif-1α pathway. Cal27, HN4, and Leuk1 cells were treated with Sal-B, knockdown of Akt or Hif-1α, the overexpression of Akt or Hif-1α, the combination of Sal-B, and overexpression of Akt or Hif-1α, respectively. Indeed, a remarkable increase of cellular proliferation (Fig. 9a, e), reduction of reactive oxygen species (ROS) production (Fig. 9b, f), and cell apoptosis (Fig. 9c, g) were demonstrated in cells with overexpressed Akt or Hif-1α, or Sal-B combination of Akt or Hif-1α overexpression. Further, JC-1 staining was used to visualize the MMP of OSCC cells, in which cells treated with either Sal-B or knockdown of Akt or Hif-1α showed a less degree of JC-1 aggregation, while overexpression of Akt or Hif-1α elicited a pronounced level of JC-1 aggregation (Fig. 9d, h and Figure S6). Our data indicated that Sal-B was able to effectively suppress MMP, ROS level, and cellular apoptosis via activation of PI3K/Akt/Hif-1α signaling.

## Discussion

Advancements in the last decade have identified altered tumor metabolism as a hallmark of cancer^14^. In contrast to normal cells that predominantly generate energy via mitochondrial oxidative phosphorylation, tumor cells rely mainly on glycolysis for energy production even in the presence of sufficient oxygen, a phenomenon termed the Warburg effect, which is the most outstanding characteristic of energy metabolism exhibited by cancer cells^14,18^. The exact mechanisms by which tumor cells primarily adopt glycolysis as their main method of energy production remains to be fully understood, but it is generally believed that aerobic glycolysis provides tumor cells with glycolytic intermediates that are evolutionarily advantageous for sustained rapid growth^18^. OSCC cells, similar to other solid tumor cells, have been demonstrated to rely primarily on aerobic glycolysis to generate the energy needed for cellular processes^18–20^. Studies have shown that glycolysis was involved with more aggressive OSCC behaviors and high levels of lactate concentration, the terminal product of glycolysis, can predict an increased incidence of metastasis formation and a poor survival in patients with OSCC^20–23^. Furthermore, the targeting of glycolysis has been validated as an increasingly intriguing novel therapeutic strategy for cancer treatment, including OSCC^24,25^.

Although it is clear that OSCC cells mainly rely on glucose catabolism through aerobic glycolysis for energy production, few reports have addressed the glycolytic dependency during the multistep process of oral carcinogenesis. Our previous study in DMBA-induced animal model for the first time revealed significant alterations of key metabolic pathways prior to cancer formation; however, it should be noted that in this work the metabolite levels were detected without measurements of the regulatory enzymes and the perturbed metabolic networks were mainly reconstructed based on the inter-metabolite correlation analysis, which might not correspond to the direct the associations between molecules^15^. Transcriptome data precluded the drawbacks and in this study revealed that compared to normal oral mucosa, significantly increased glucose metabolism was indeed observed in premalignant dysplasic oral tissues and persisted as disease progression, suggesting that glycolytic switch had a pivotal role in early pathogenesis of oral carcinogenesis and rewiring to rely on glucose catabolism through aerobic glycolysis for energy production may be a critical selection force in malignant transformation from a premalignant lesion to OSCC^26^. The finding that glycolytic dependency occurred in preneoplastic lesions promoted us that targeting aberrant glycolysis might be suitable for chemopreventive interventions prior to OSCC transformation.

Sal-B derived from Chinese herbs has been shown to have therapeutic potential toward the prevention of oral cancer initiation and progression, whereas few reports have addressed the effect of Sal-B on OSCC cellular metabolism. In this study, Sal-B was demonstrated to cause downregulation of the elevated glucose metabolism during oral carcinogenesis, which was consistent with the chemopreventive effect of Sal-B in this animal study. Metabolism modulation should be an additional mechanism involved in anti-carcinogenic action of Sal-B. Deciphering the molecular mechanisms or regulatory pathways that contribute to metabolic rewiring in specified settings has the potential to facilitate the development of the glycolysis-based therapeutic interventions for cancer^27^. Transcriptome data in this study suggested that Sal-B exerted anti-carcinogenic effects by inhibiting the Warburg effect, which was mainly through the potentially oncogenic PI3K/Akt/HIF-1α signaling pathways. For validation, over-activation of the key proteins in PI3K/Akt/HIF-1α signaling pathways were detected in dysplasic lesions and cancers from hamsters and Sal-B treatment significantly inhibited the signaling pathway that is constitutively activated in human OSCC^28^. We further successfully verified the results in two well-recognized OSCC cell lines and one potentially malignant Leuk1 cell line. Sal-B treatment can inhibit glycolysis and activation of key proteins of PI3K/Akt/HIF-1α signaling pathways. The PI3K/Akt pathway lies downstream of receptor tyrosine kinase activation and is involved in many cellular processes, such as inflammation, motility, autophagy, and cancer progression^29,30^. Besides, it serves an important role in the tightly controlled regulation of metabolic adaptations that support cell growth^29,31^. Akt as a downstream effector of PI3K is known as an important driver of the tumor glycolytic phenotype and renders cancer cells dependent on glycolysis for survival^31^. Akt stimulates glucose uptake and glycolysis by increasing the expression and membrane translocation of glucose transporter proteins like GLUT1 and by activating glycolytic enzymes and regulating HK expression, activity and interaction with mitochondria^30^. The most dramatic metabolic consequence of PI3K/Akt activation is downstream activation of the metabolic checkpoint kinase complex mTOR (mTORC1 and mTOR2)^30,32^. mTOR induce transcription of many genes involved in aerobic glycolysis and tumor growth, including HIF-1α, nuclear factor-κB, and c-Myc, among which HIF-1a was previously demonstrated to be inhibited by Sal-B treatment^11,30,32^. HIF-1a is a key molecule in the regulation of hypoxia and tumor glycometabolism. Indeed, in our study, we identified that anti-tumor role of Sal-B might be partially contributed by productive modulation of MMP, ROS level, and cellular apoptosis via inhibiting HIF-1α signaling pathway. Knockdown of HIF-1α showed significantly reduction of intracellular glucose uptake. On the other hand, the overexpression of HIF-1α led to a remarkable increment of glucose uptake. Either HIF-1α knockdown induced a significantly reduced level of intracellular lactate and the lactate secretion into supernatant. The knockdown of HIF-1α led to the inhibition of cellular proliferation, the increase of ROS production, and apoptosis, as well as decreased MMP. These results has unraveled regulatory pathways on metabolism modulation function of Sal-B as key mechanisms of action of Sal-B, which is critical from a therapeutic perspective and merits further investigation in the preclinical setting before embarking on clinical trials.

**Fig. 10:**
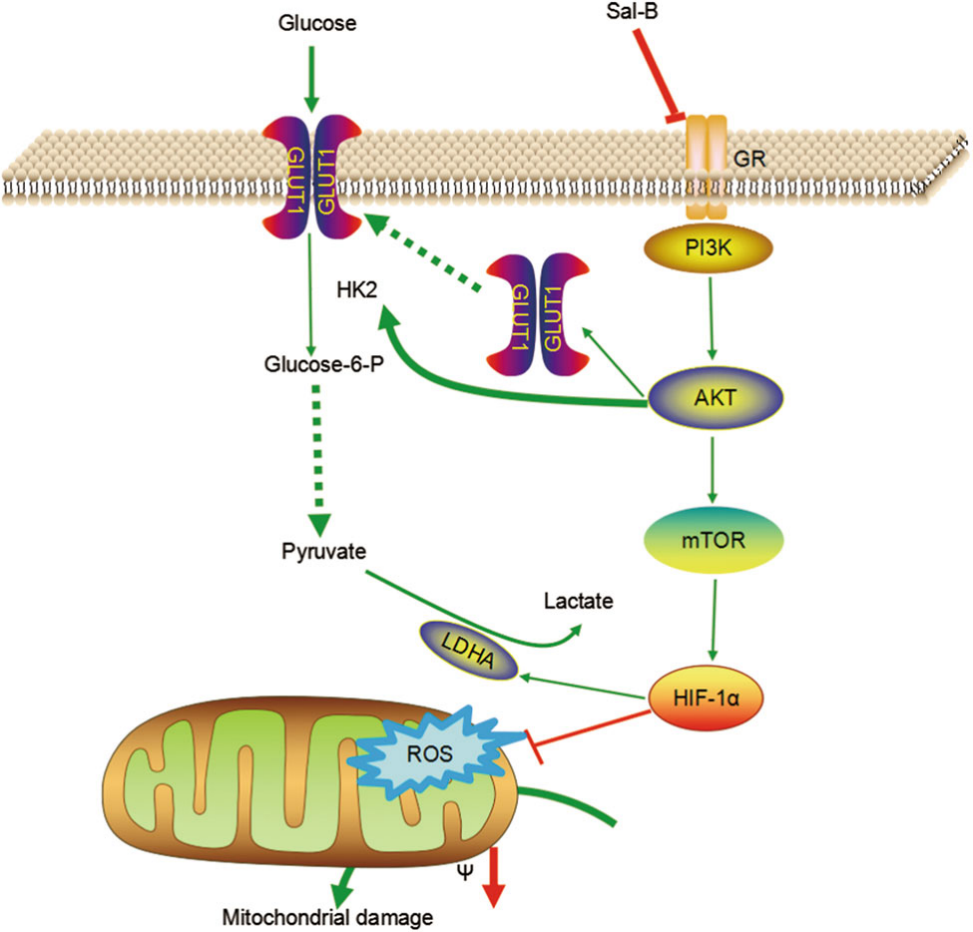
Schematic model of Sal-B in the Warburg effect and anti-carcinogenic activity. GLUT1 primarily transports glucose and HK2 catalyzes the first step of glycolysis by conversion of glucose into glucose-6-phosphate and the terminal product of glycolysis is lactate. GLUT1 and HK2 are induced by activated PI3K and HIF-1α signaling pathways. These processes can be blocked by Sal-B treatment, which was attributable to the anti-carcinogenic activity of Sal-B

Mitochondrion is an important intracellular organelle, with a crucial function in ATP generation, which is reliant on the stability of MMP. Loss of MMP would be predicted to have reduced oxidative phosporylation and ROS production^30^. In the present study, these cells exposed to various concentrations of Sal-B resulted in dissipation of MMP, which might lead to oxidative phosphorylation stops to limit the production of ROS-mediated damage and eventually contribute to OSCC cell apoptosis. Above all, we have summarized a schematic depicting the proposed mode of action of Sal-B in the Warburg effect and anti-carcinogenic activity based on the results from this study (Fig. 10). GLUT1 primarily transports glucose and HK2 catalyzes the first step of glycolysis by conversion of glucose into glucose-6-phosphate and the terminal product of glycolysis is lactate. Proliferating oral mucosa cells rely primarily on aerobic glycolysis to generate the energy needed for cellular processes. GLUT1 and HK2 are induced by activated PI3K/AKT/HIF-1α signaling pathways. These processes can be blocked by Sal-B treatment, which are attributable to the anti-carcinogenic activity of Sal-B.

This study has for the first time demonstrated that Sal-B inhibits the oncogenic PI3K/AKT/HIF-1α signaling pathways that are activated in DMBA-induced model and modulates the aberrant glucose metabolism to block the malignant transformation, despite whether it was a causal relationship warrants further studies. Tumors are generally typified by phenotypic diversity and a single model of altered tumor metabolism generally does not describe the sum of metabolic changes that can support cell growth. It would be interesting in the future to unravel the metabolic heterogeneity and flexibility that will contribute towards the chemopreventive effect of Sal-B on OSCC cells.

## Materials and methods

### Animal treatment and sampling

The carcinogenic effects of DMBA and the anti-carcinogenic effects of Sal-B have been verified by other and our institutes. In this study, we established the animal study according to our published protocol approved by Xin Hua Hospital, affiliated to Shanghai Jiao Tong University School of Medicine. Brifely, 6–8 weeks old male Syrian hamsters weighting between 60 and 80 g were obtained from Shanghai Song lian Laboratory Animal Co. Ltd., China. The animals were housed five per cage in a room with controlled temperature and humidity with 12 h light–dark cycles. After 1 week of acclimatization, the hamsters were randomly divided into three groups: DMBA-treated model group (*n* = 35), the left pouches of hamsters were topically treated with 100 ml of 0.5% DMBA (in acetone) solution three times/week for 9 weeks; normal control group (*n* = 15), the left pouches of hamsters were topically treated with 100 ml water as processed in DMBA-induced model group; Sal-B-DMBA-treated group (n = 35), a daily dose of 10 mg/kg of body weight Sal-B was given in a 100 ml aliquot via oral gavage and painting the pouch with 100 μl of 0.5% DMBA solution three times/week for 9 weeks. At the end of 4th, 5th, 6th, 7th, and 9th week, 3−12 hamsters were killed and tissue samples were collected.

### Hematoxylin and eosin staining

Cheek pouches were collected, fixed with formalin, embedded in paraffin, and serially sectioned at a 2 μm thickness. Sections were histopathologically analyzed by hematoxylin and eosin staining. Dysplasia and carcinoma were diagnosed according to our previous published work, in which dysplasia was characterized by irregular epithelial stratification, increased number of mitotic figures, increased nuclear-tocytoplasmic ratio, and loss of polarity of basal cells and carcinoma was featured with the invasion of underlying tissues.

### Reagents and antibodies

Sal-B (purity > 99%) was obtained from the Chinese National Institute for the Control of Pharmaceutical and Biological Products (Beijing, China). Antibodies for PI3K, protein kinase B/Akt, p-Akt, mTOR, phospho-mTOR, and HIF-1α were purchased from Cell Signaling Technology (Boston, USA). Antibodies against GLUT1 and HK2 were purchased from Abcam (Cambridge, Britain). Rabbit anti-glyceraldehyde-3-phosphate dehydrogenase (GAPDH) antibody was purchased from Sigma-Aldrich (San Francisco, USA).

### Transcriptomic profiling of the rat model

Eight representative cheek pouches excised from normal control group, DMBA-induced model group (histopathologically sub-divided into two groups, designated as dysplasia and cancer group), and Sal-B-DMBA-treated group were selected for second-generation high-throughput sequencing (two/four groups). Briefly, total RNA was extracted using TRIzol reagent. The quality and intergrity of RNA samples were measured with a NanoDrop 2000 spectrophotometer. Library construction and sequencing were carried out by Suzhou GENEWIZ institute. Total RNA samples were first enriched and purified for mRNA, followed by sonication and reverse transcription. Sequencing was launched on the Illumina HiSeq2000 platform and read quality was assessed using FastQC. Reads of poor quality were filtered according to standard Illumina criteria and the remaining clean reads were aligned to the reference mesocricetus auratus genomic profiles.

Gene expression was quantified by default using HTSeq-Count (Version 0.6.1). Differential gene expression was performed using DESeq2 (Version 1.2.10). Transcripts with a fold change > 2 with adjusted *p*-value < 0.05 were considered significant DEGs. Comparisons between each group were performed. GO analyses were applied to analyze the main function of DEGs according to the GO database, which provided high-quality electronic and manual annotations for genes. Pathway analyses were used to determine the significant pathways of DEGs according to the KEGG, which provided a global map of biological systems. Gene interaction networks were constructed to indicate the underlying interactions of genes and the networks were clustered into modules and pathways enriched in the modules were identified. Omicsbean-cancer was used for data analysis (http://www.omicsbean-cancer.com). For each category, the Pearson’s *χ*^2^-test and two-tailed Fisher’s exact tests were employed and *p* < 0.05 was considered statistically significant.

### Cell lines and cell culture

Human OSCC cell line Cal27 and LN4 were obtained from Laboratory of Oral Tumor and Oral Biology, Shanghai Key Laboratory of Stomatology, Shanghai, China. Leuk1 was a premalignant leukoplakia cell line kindly provided by Professor Li Mao of University of Maryland Dental School, Baltimore, USA. Cells were maintained in a humidified atmosphere containing 5% CO_2_ at 37 °C. Leuk1 and Cal27 cells were cultured in Dulbecco’s modified Eagle’s medium (DMEM) (GIBCO, USA) medium supplemented with 10% fetal bovine serum (FBS) (GIBCO). LN4 were cells were maintained in DMEM/F12 (GIBCO) supplemented with 10% FBS (GIBCO).

### Western blot analysis

The procedures for preparation of cell or tissue protein lysates for western blotting were modified from previously published protocols. Briefly, protein lysates (15 μg) of total protein were electrophoretically separated on 10% denaturing polyacryamide gels and transformed to polyvinylidene difluoride membranes using a semidry blotting technique according to the manufacturer’s protocol. The membranes were blocked with 5% non-fat milk and probed with antibodies against the specified proteins. The same blots were used for probing phospho-specific antibodies and antibodies against total protein. GAPDH was used as loading controls.

### Measurement of glucose uptake and lactate secretion

Cells were treated with different concentrations of Sal-B for 48 h, medium were collected, and diluted within water. Glucose uptake was determined by subtracting the amount of glucose in each sample from the total amount of glucose in the medium according to the instruction book of Glucose Assay Kit (Rsbio, China). Besides, medium were assayed by Lactic Acid Production Detection kit (KeyGen, China) following the operation instructions to measure the generation of lactic acid. Assays were evaluated by a colorimetric reaction (absorbance at 530 nm).

### Measurement of MMP

As an index to determine mitochondrial dysfunction, MMP was monitored using JC-1, a specific probe to mitochondria. Briefly, cells were treated with Sal-B at indicated concentration for 48 h in a six-well culture plate at 1 × 10^5^ cells/ml. The media were removed and washed three times with serum-free DMEM medium followed by incubation in fresh serum-free medium containing 5 mg/l JC-1 at 37 °C in dark for 10 min, and washed with phosphate-buffered saline (PBS) for three times. Then MMP was identified by calculation of the ratio of JC-1 ploymer to monomer using fluorecence microscopy.

### Assessment of apoptosis

TUNEL assays were performed to detect apoptotic cells in OSCC cells according to the manufacturer’s protocol. Briefly, cells were treated with Sal-B at indicated concentration for 48 h in a six-well culture plate at 1 × 10^5^ cells/ml. Cells were washed with PBS and fixed with the buffer supplied in the kit. Cell nucleus were stained with DAPI (4′,6-diamidino-2-phenylindole) at 25 °C for 10 min. TUNEL-positive cells were defined as those with dark green fluorescent staining and these were identified via fluorescence microscopy. To quantify TUNEL-positive cells, the number of green fluorescence-positive cells was counted in four to six random fields at × 200 magnification.

### MTT assay

The effect of Sal-B on cell viability was measured by MTT colormetric method. Cells were prepared at a density of 5 × 10^4^/ml after trypsinization and inoculated into a 96-well culture plate (100 μl per well). The liquid was discarded after 12 h culturing, while an increasing dose of Sal-B were added. After drug treatment for 18–48 h, attached cells were incubated with MTT (0.5 mg/ml) for 4 h and subsequently 150 μl dimethylsulfoxide was added. The absorbance of each well was measured using an enzyme-linked immunosorbent assay reader at 490 nm. Each group had six parallel complex holes and experiments were performed at least three times. The inhibition rates (%) were calculated as follows: cell inhibition rate (%) = 1 − (*D*_the value of experimental group_ − *D*_the value of blank control group_)/(*D*_the value of control group_ − *D*_the value of blank control group_) × 100%.

### Clone formation assay

Cells were seeded in six-well plates with 5 × 10^2^ cells per well and cultured at 37 °C with 5% CO_2_ for 14 days. The cell colonies were washed twice with PBS, fixed in 4% paraformaldehyde for 30 min, and stained with Giemsa for 20 min. Individual colonies with more than 50 cells were counted under a microscope. Clone formation ratio (%) = clone number/planted cell number by 100%.

### AKT or Hif stable overexpressing or knockdown cell establishment

The AKT or Hif-1a overexpression or knockdown lentivirus were purchased from Genepharma Company (China). The cells were infected with lentivirus and selected in puromycin (Invitrogen, USA). The clones were confirmed by western blotting. Stable cell lines were maintained in DMEM supplemented with 10% FBS and 2 μg/ml puromycin.

### Statistical analysis

All data are presented as the mean ± SD of at least three triplications. Parametric data in different groups were examined for the homogeneity of variance and the data with equal variances were compared using one-way analysis of variance followed by least significant difference method for the inter-multiple group comparisons; non-parametric data or data with unequal variances were compared using Kruskal–Wallis rank test followed by Dunnett T3 method for inter-multiple group comparisons (GraphPad Software, San Diego, CA). Significant differences of parametric data between two groups were determined by two-tailed unpaired Student’s *t*-test. Non-parametric data in two groups were determined by Mann–Whitney *U*-test. *P* < 0.05 was considered a statistically significant difference.

## Electronic supplementary material

supplementary Figures

sTable1

sTable2

sTable3
