# Supplementary material for: Salvianolic acid B inhibits glycolysis in oral squamous cell carcinoma via targeting PI3K/AKT/HIF-1α signaling pathway
Source: Cell Death Dis. 2018 May 22;9(6):599. doi: 10.1038/s41419-018-0623-9 (PMC5964095; doi:10.1038/s41419-018-0623-9)
Supplement: Supplementary file 1 — supplementary Figures [file 41419_2018_623_MOESM1_ESM.doc]

**Supplementary figures**


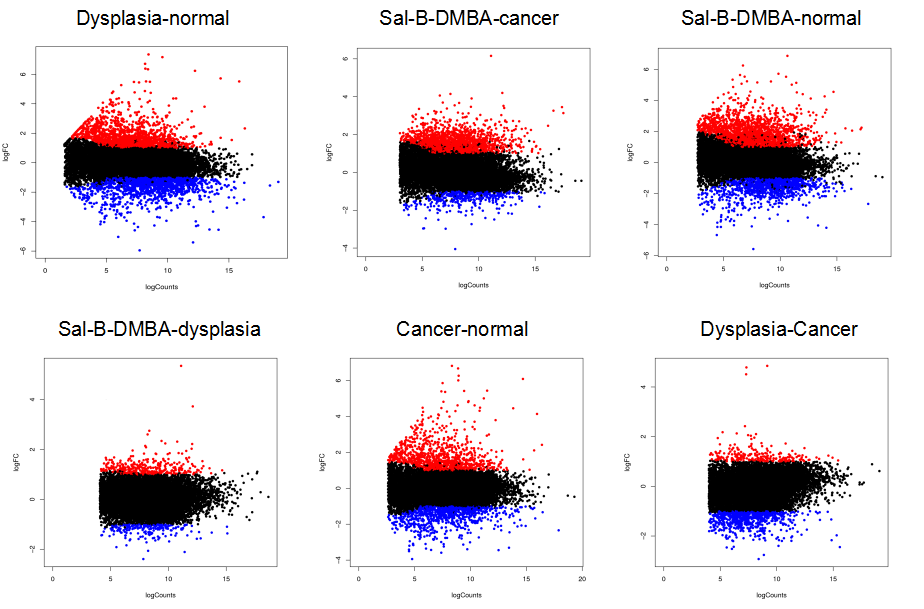


**Figure S1: Six pairwise, matched-sample comparisons were shown.** Six volcano figures were presented. Each volcano plot shows that the two vertical lines are the 2-fold change boundaries and the horizontal line is the statistical significance boundary (p<0.05). Genes with fold change≥2 and statistical significance are marked with red dots.


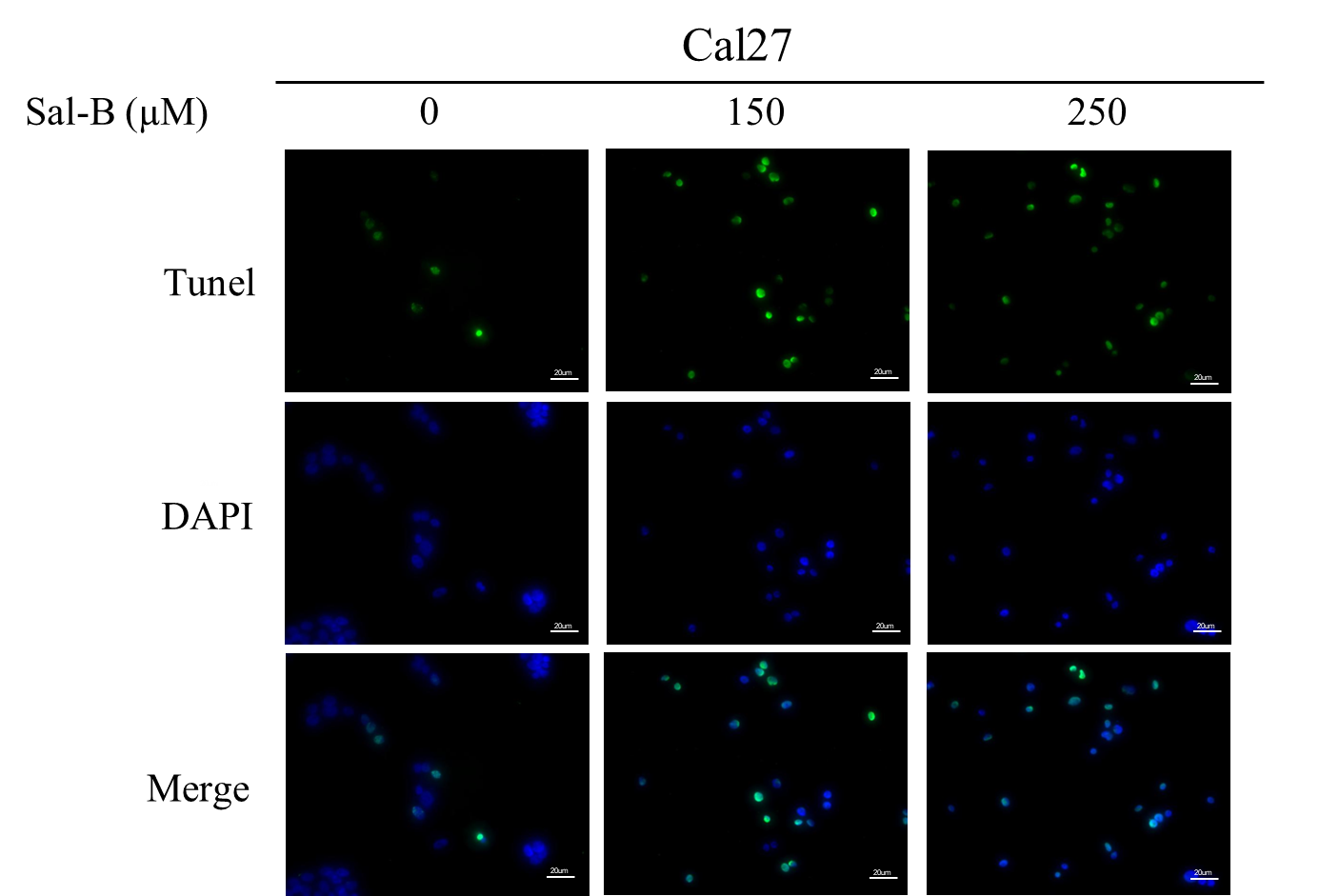


（A）


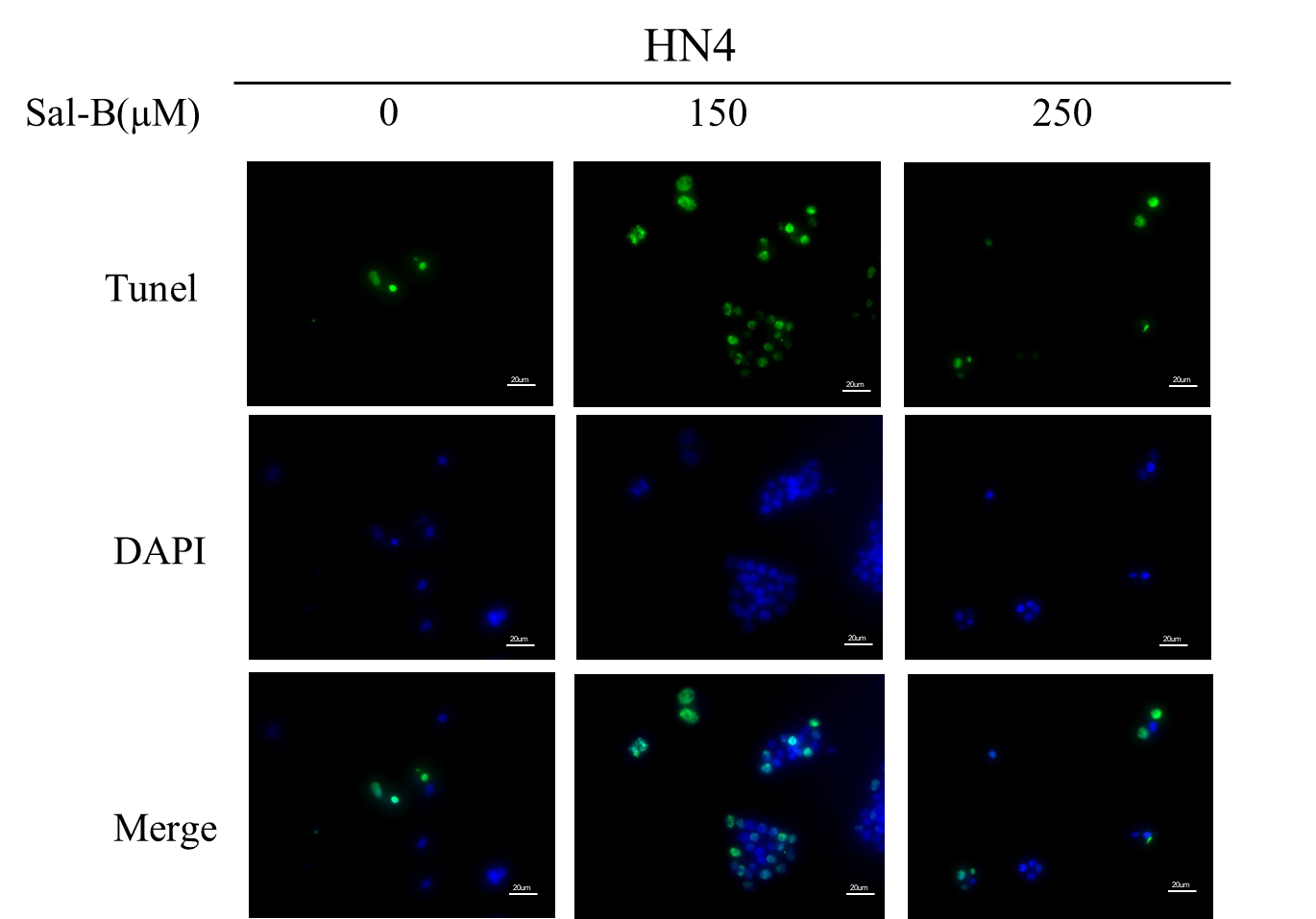


(B)


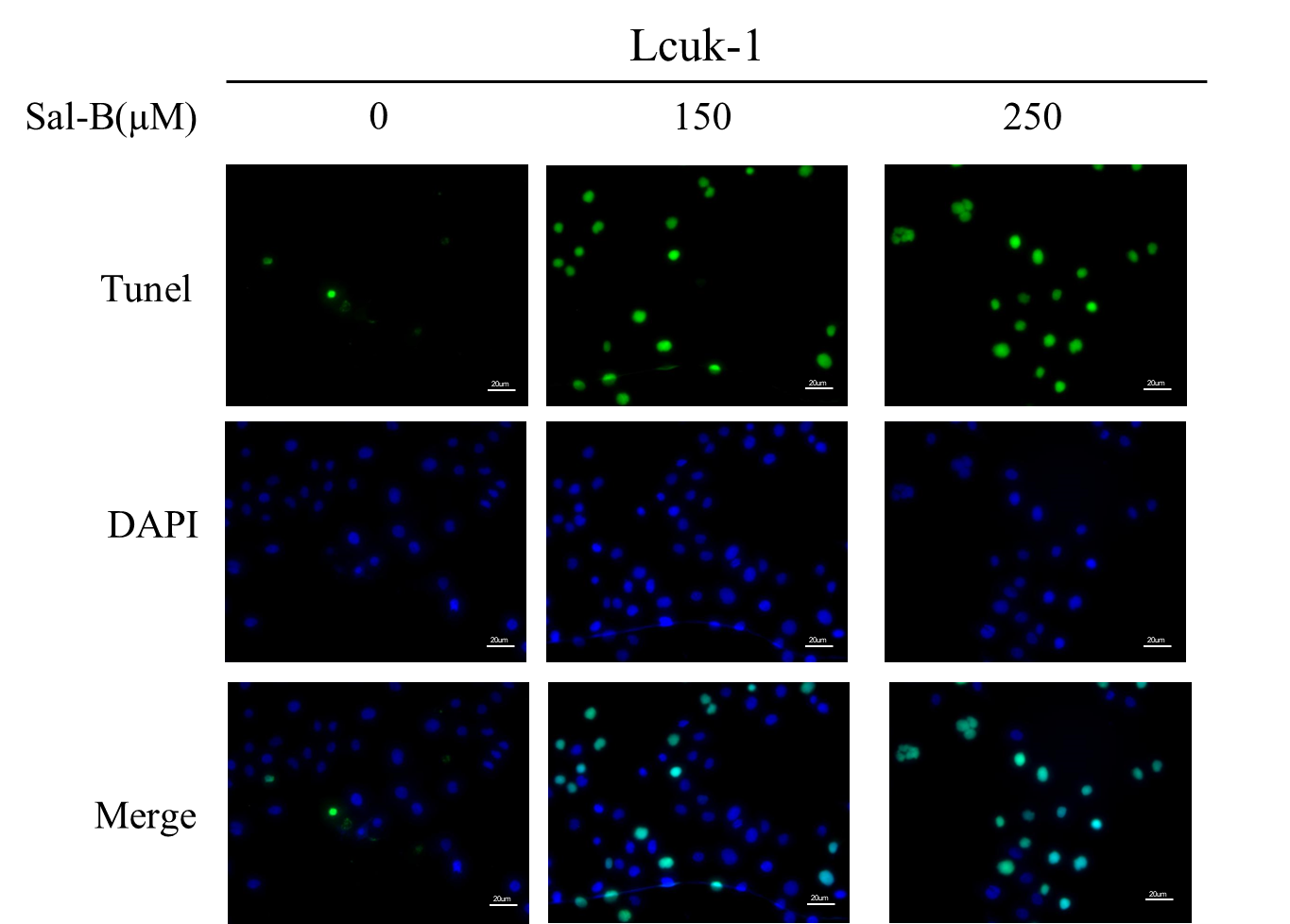


(C)

**Figure S2 Apoptosis of Cal27, HN4 and Leuk-1 cells with treatment of 0, 150 and 250 uM of Sal-B measured by TUNEL assay.** Bar stands for 20 μm.


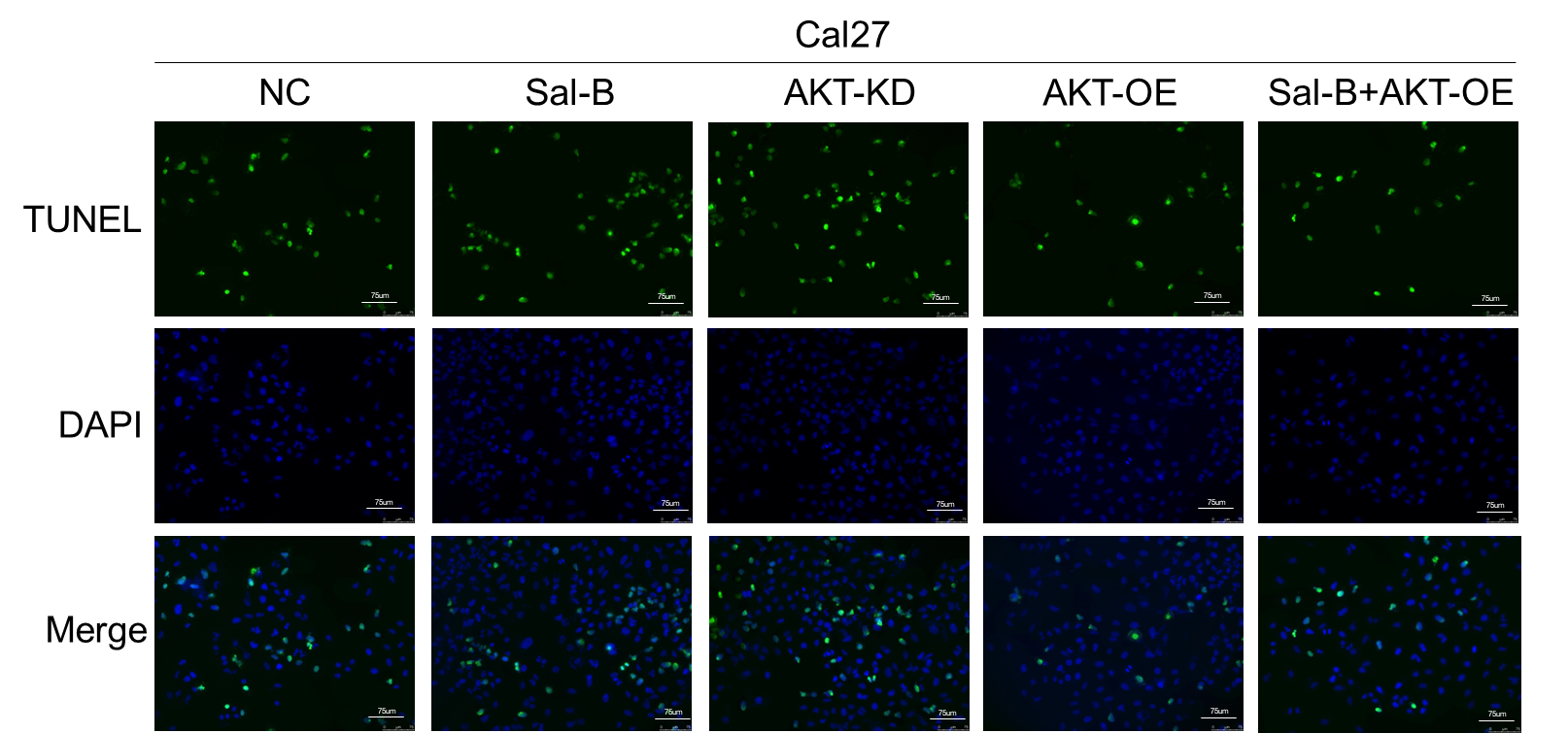


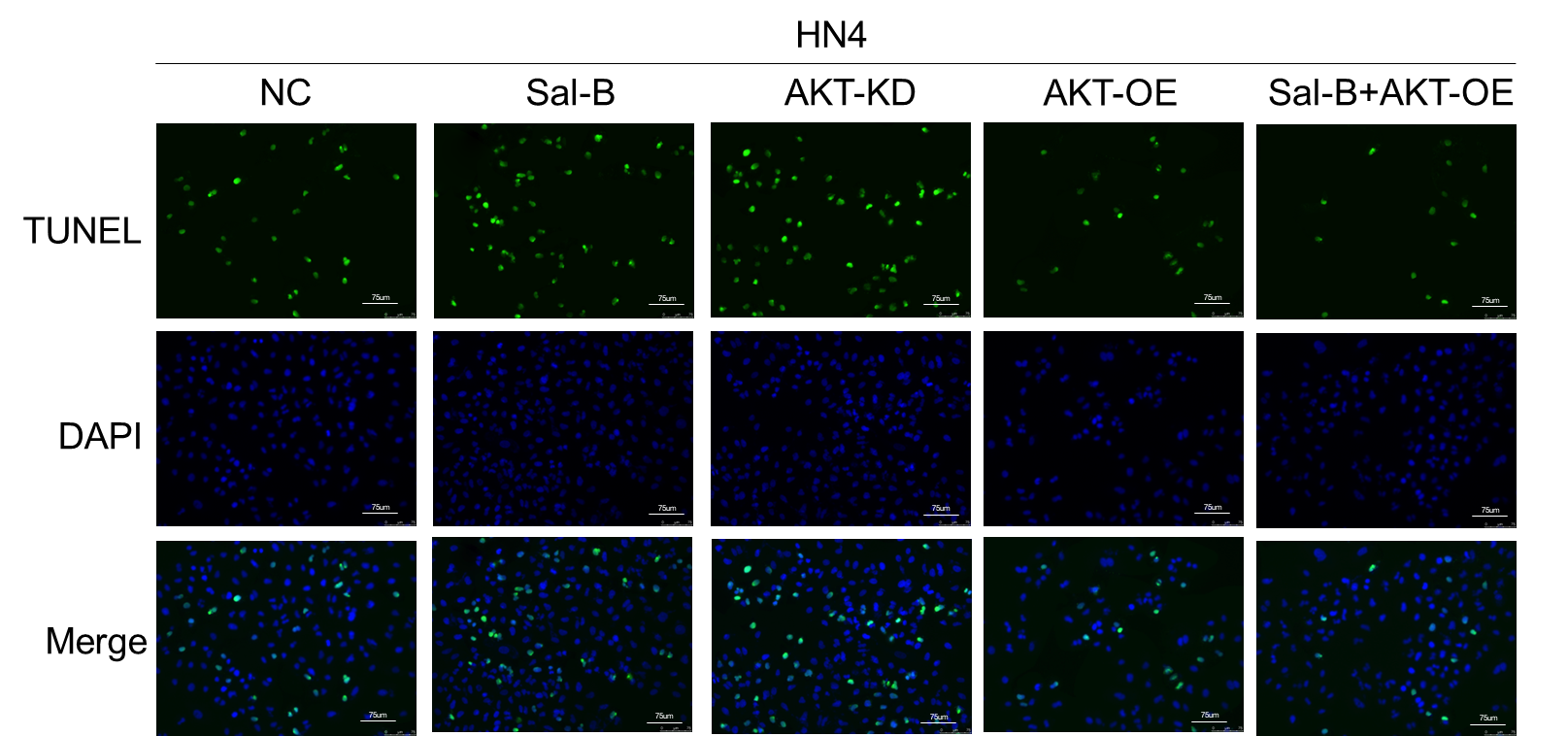


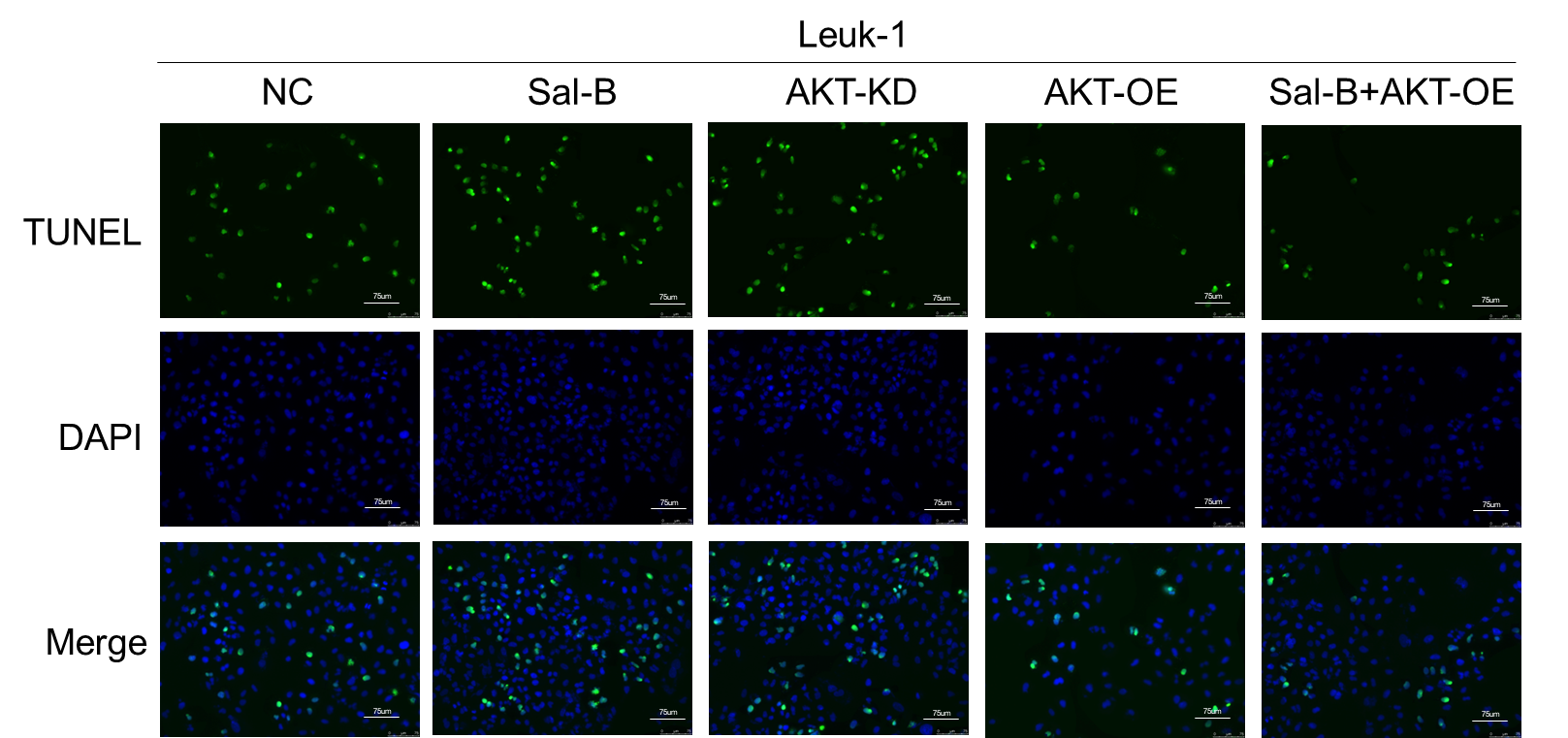


(C)

**Figure S3 Apoptosis was detected by TUNEL assay.**

Apoptosis of Cal27 (A), HN4 (B) and Leuk-1(C) cells were treated with varying conditions, including (1) without treatment (NC), (2) 250 uM of Sal-B, (3) knockdown of AKT expression using AKT shRNA lentivirus (AKT-KD); (4) overexpression of AKT using transfection of AKT lentivirus (AKT-OE); (5) the combined treatment of Sal-B and AKT-OE. Bar stands for 75μm.


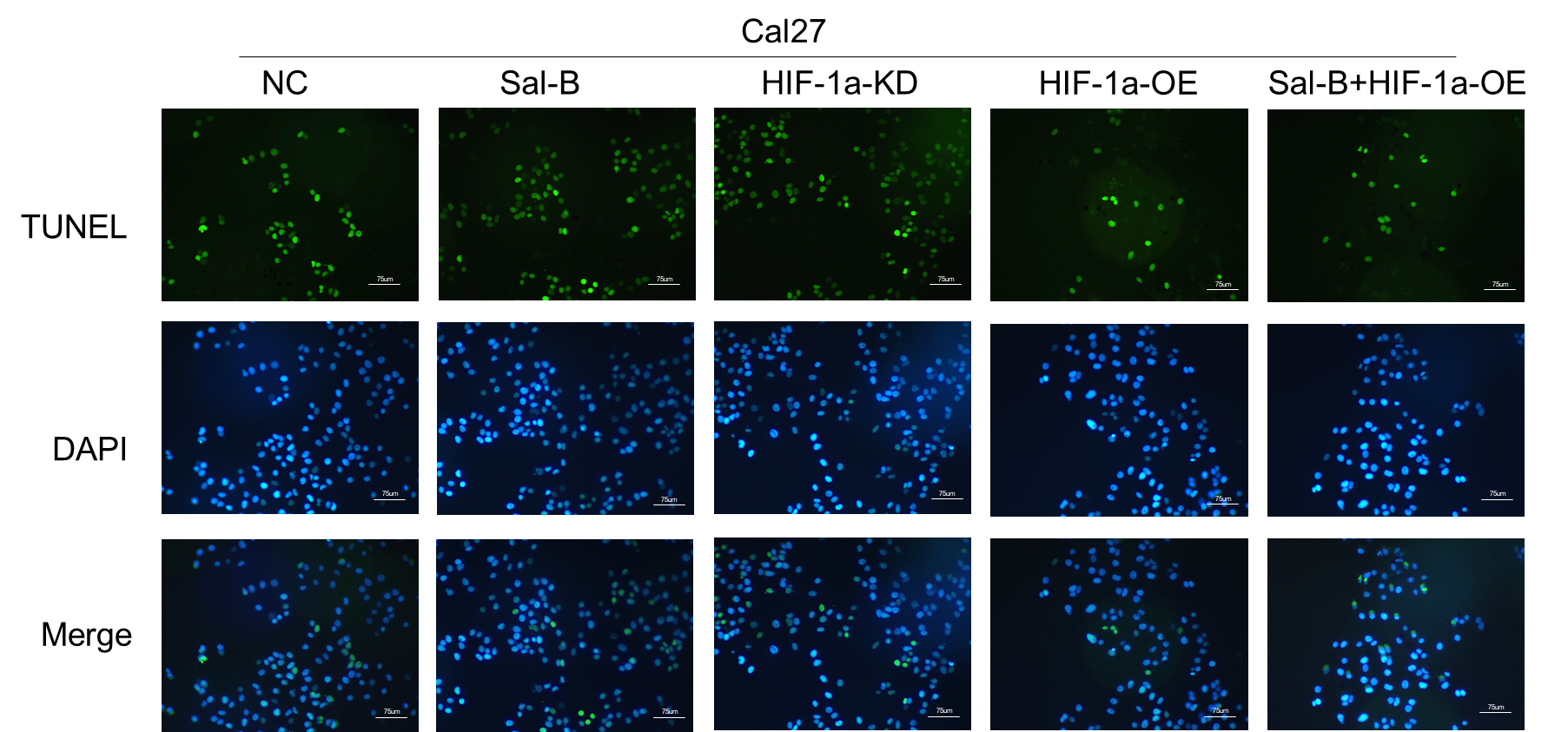


(A)
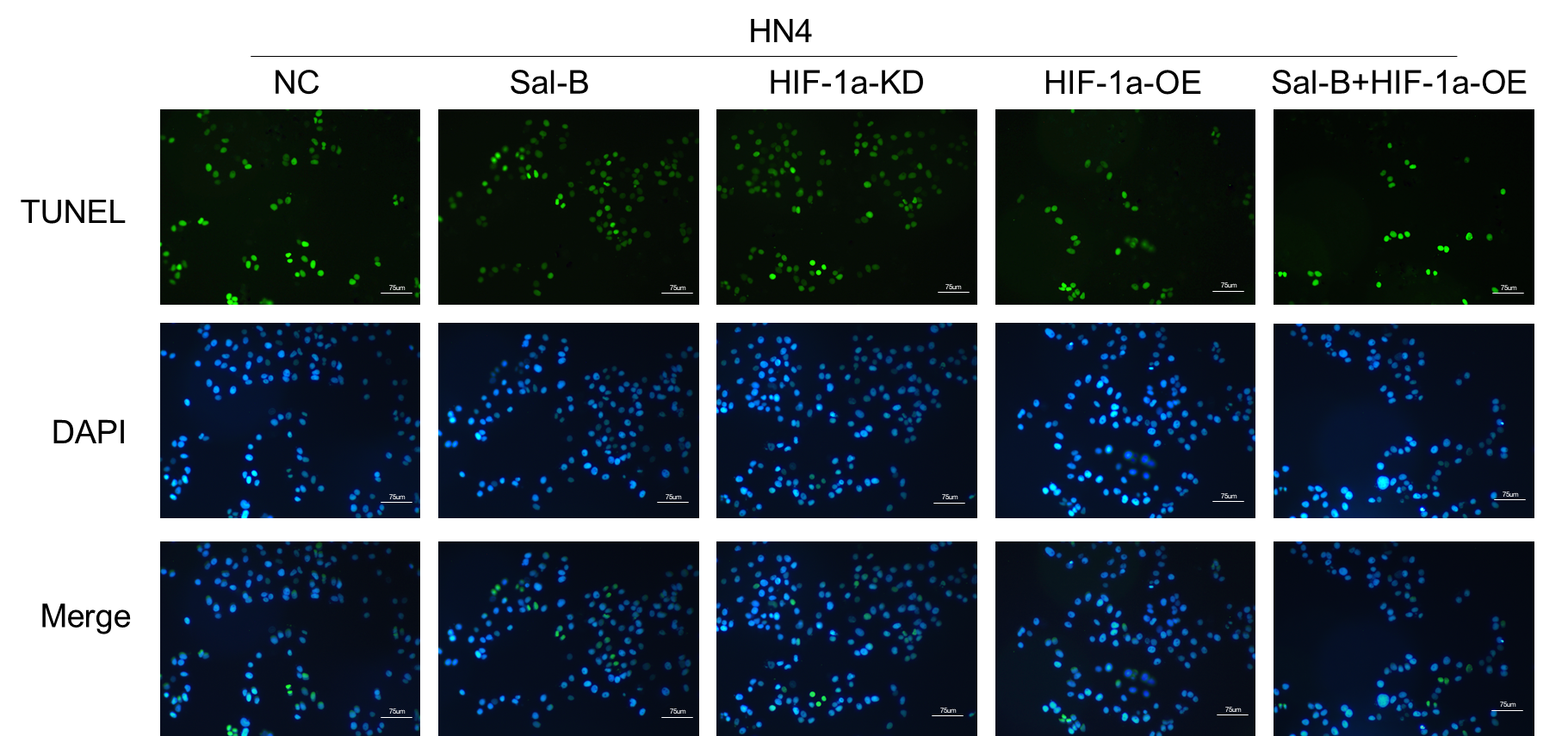


(B)


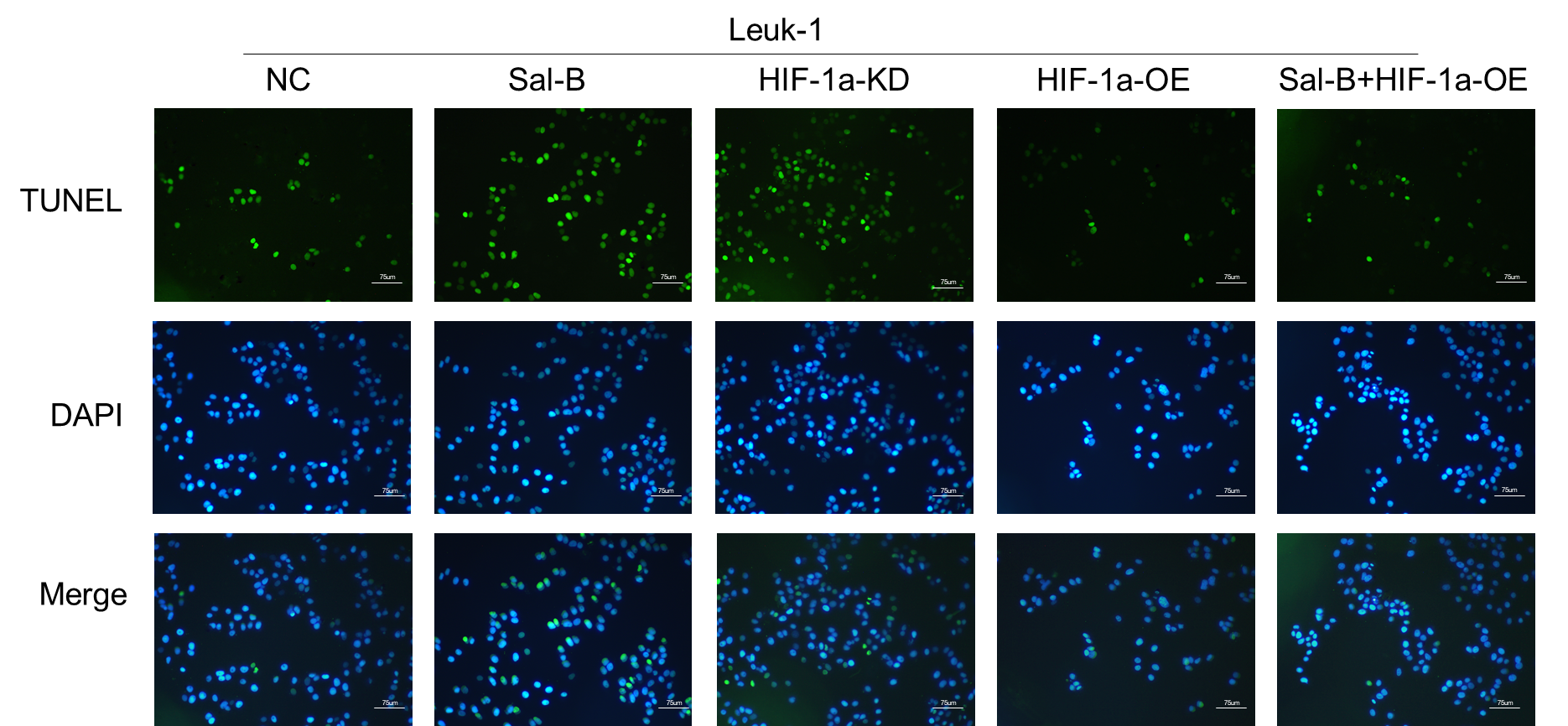


(C)

**Figure S4 Apoptosis was detected by TUNEL assay.**

Apoptotic cell death of Cal27 (A), HN4 (B) and Leuk-1(C) cells were treated with varying conditions, including (1) without treatment (NC), (2) 250 uM of Sal-B, (3) knockdown of HIF-1α expression using Hif-1α shRNA lentivirus (HIF-1α-KD); (4) overexpression of HIF-1α using transfection of Hif-1α lentivirus (HIF-1α-OE); (5) the combined treatment of Sal-B and HIF-1α. Bar stands for 75μm.

Supplementary figure 5


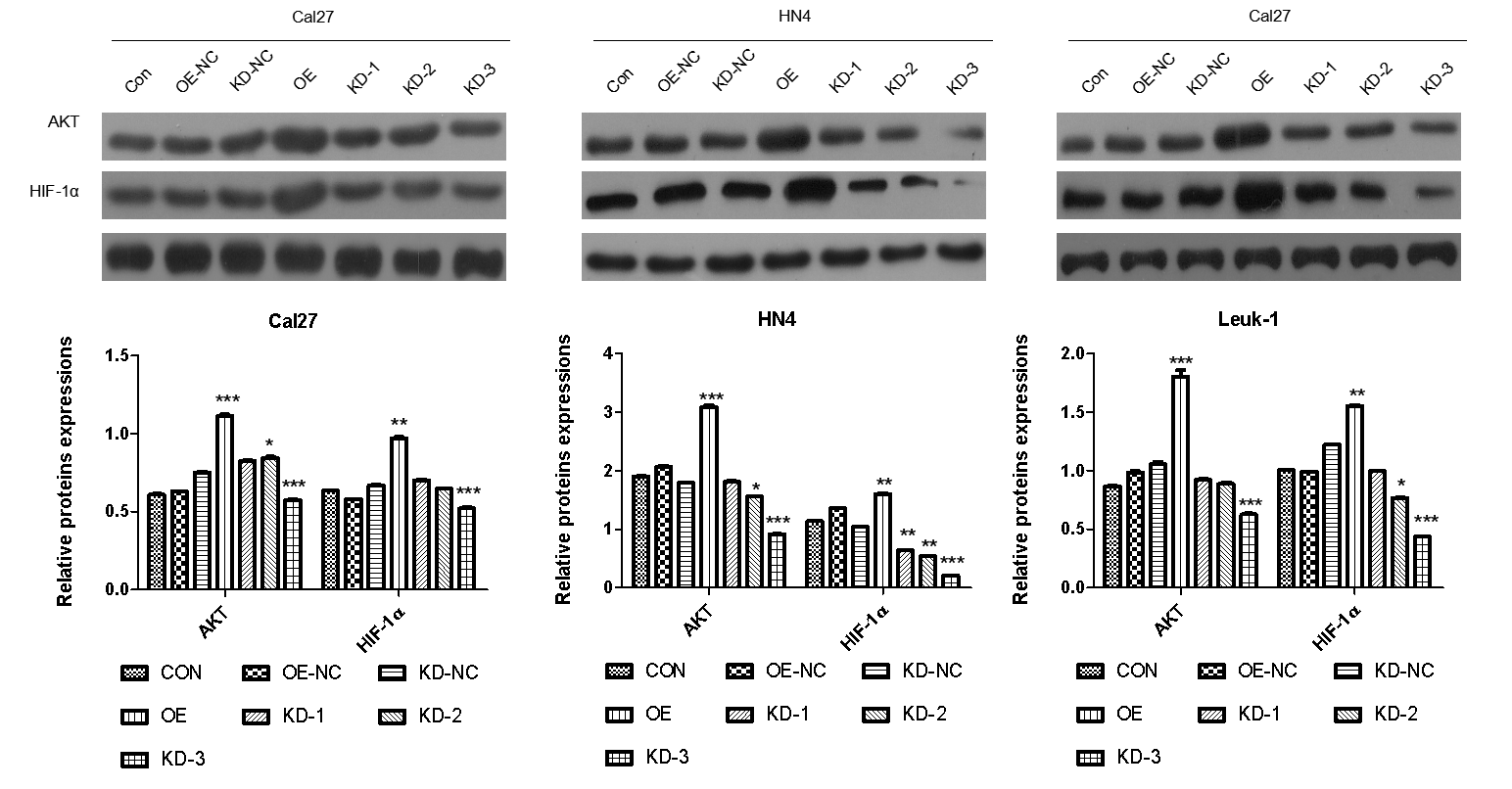


(A)


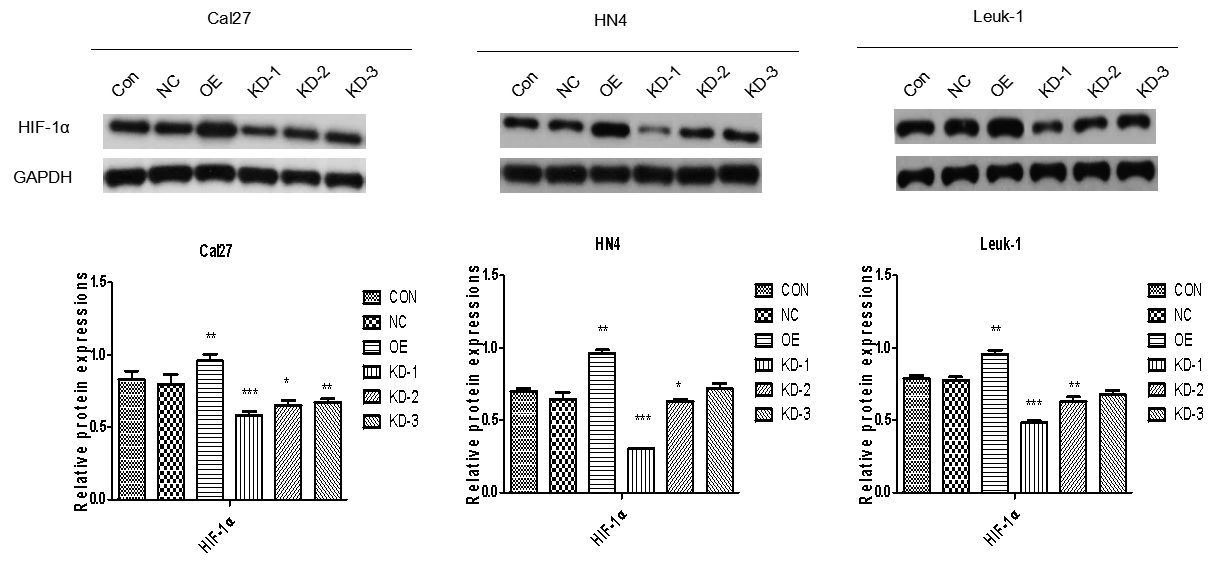


(B)

**Figure S5 Western blot detecting the overexpression or knockdown of AKT or Hif-1α**

1. Western blot detecting the overexpression or knockdown of AKT.
2. Western blot detecting the overexpression or knockdown of Hif-1α.


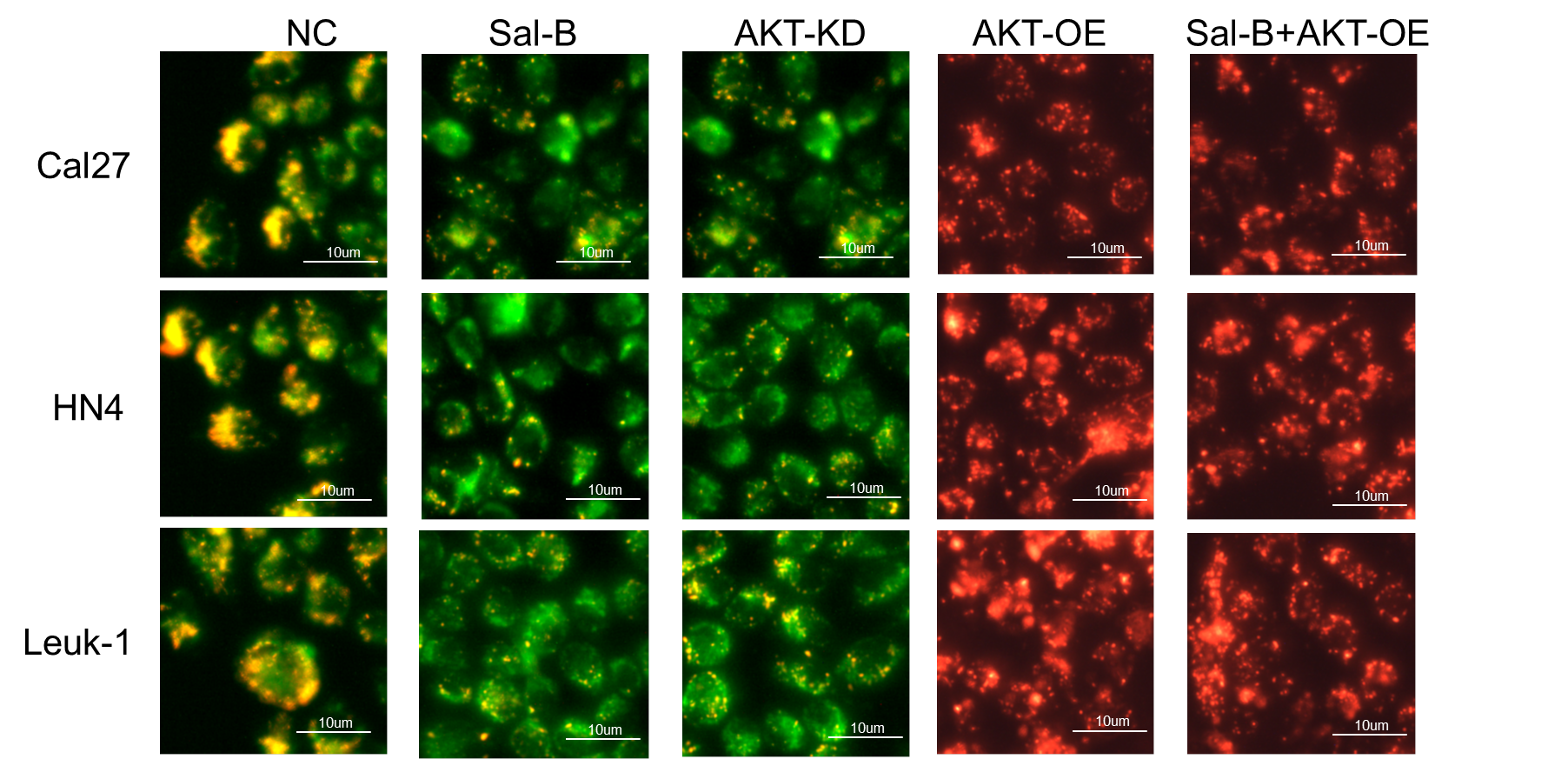


(A)


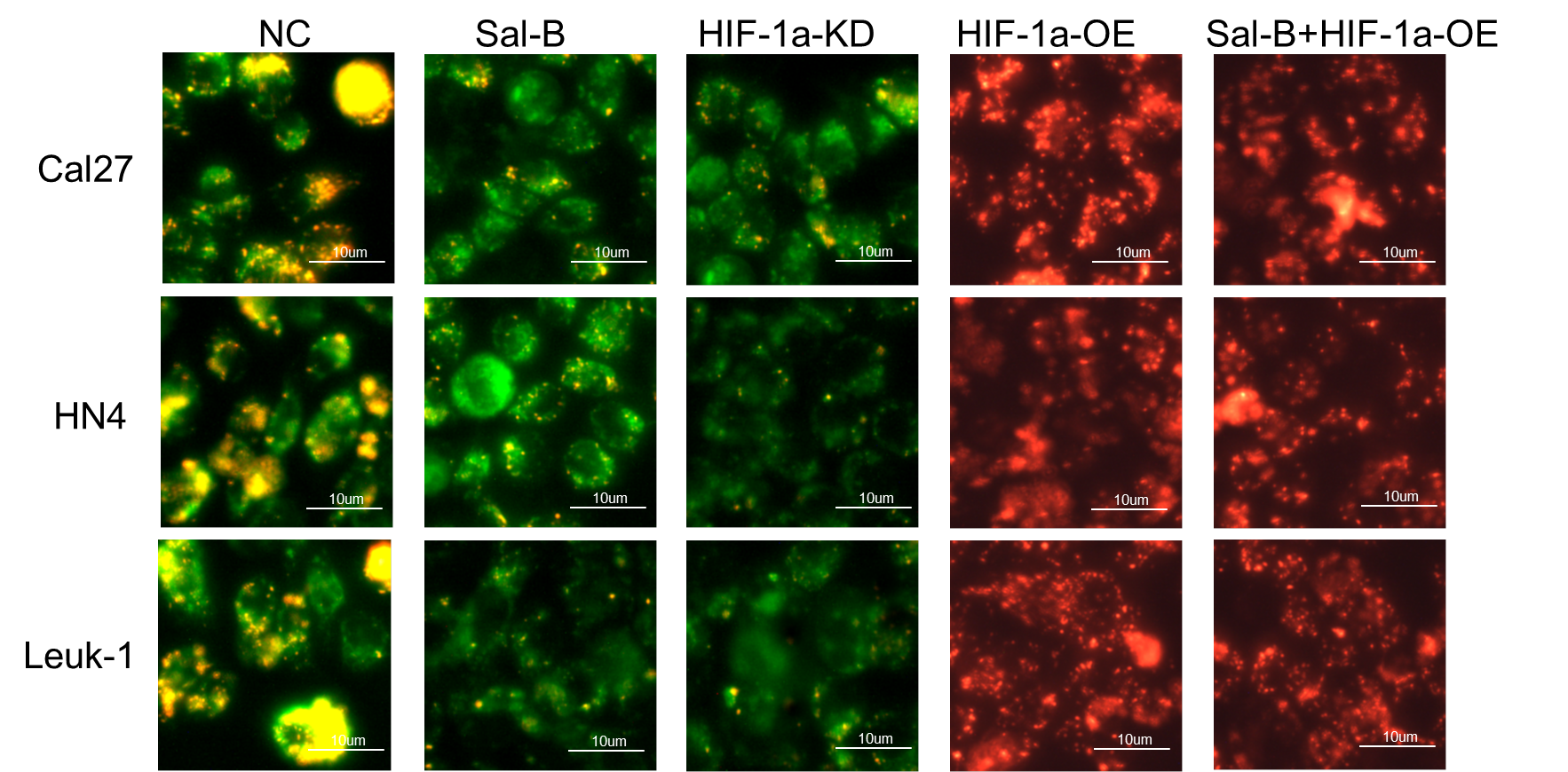


(B)

**Figure S6 MMP was measured by JC-1 staining**

(A) MMP was measured by JC-1 staining in Cal27, HN4 and Leuk1 cells under varying conditions described in varying conditions, including (1) without treatment (NC), (2) 250 uM of Sal-B, (3) knockdown of AKT expression using AKT shRNA lentivirus (AKT-KD); (4) overexpression of AKT using transfection of AKT lentivirus (AKT-OE); (5) the combined treatment of Sal-B and AKT-OE. Bar stands for 10 μm

(B) MMP was measured by JC-1 staining in Cal27, HN4 and Leuk1 cells under varying conditions described in varying conditions, including (1) without treatment (NC), (2) 250 uM of Sal-B, (3) knockdown of HIF-1α expression using Hif-1α shRNA lentivirus (HIF-1α-KD); (4) overexpression of HIF-1α using transfection of Hif-1α lentivirus (HIF-1α-OE); (5) the combined treatment of Sal-B and HIF-1α. Bar stands for 10 μm.
